# Extracellular Vesicle Transfer from Endothelial Cells Drives VE-Cadherin Expression in Breast Cancer Cells, Thereby Causing Heterotypic Cell Contacts

**DOI:** 10.3390/cancers12082138

**Published:** 2020-08-01

**Authors:** Maryam Rezaei, Ana C. Martins Cavaco, Martin Stehling, Astrid Nottebaum, Katrin Brockhaus, Michele F. Caliandro, Sonja Schelhaas, Felix Schmalbein, Dietmar Vestweber, Johannes A. Eble

**Affiliations:** 1 Institute of Physiological Chemistry and Pathobiochemistry, University of Münster, 48149 Münster, Germany; mar.rezaei80@gmail.com (M.R.); acmcavaco@gmail.com (A.C.M.C.); kat.brock@gmx.de (K.B.); caliandromichelefabrizio@gmail.com (M.F.C.); Felix_Schmalbein@web.de (F.S.); 2 Luis Costa Lab, Instituto de Medicina Molecular, Faculdade de Medicina da Universidade de Lisboa, 1649-028 Lisboa, Portugal; 3 Department of Cell and Developmental Biology, Flow Cytometry Unit, Max Planck Institute for Molecular Biomedicine, 48149 Münster, Germany; martin.stehling@mpi-muenster.mpg.de; 4 Department of Vascular Cell Biology, Max Planck-Institute of Molecular Biomedicine, 48149 Münster, Germany; a.nottebaum@mpi-muenster.mpg.de (A.N.); vestweb@mpi-muenster.mpg.de (D.V.); 5 European Institute for Molecular Imaging (EIMI), University of Münster, 48149 Münster, Germany; sonja.schelhaas@uni-muenster.de

**Keywords:** breast cancer, human umbilical vein endothelial cells (HUVEC), epithelial-mesenchymal transition (EMT), cadherin switching, extracellular vesicles (EVs), invasion, vascular mimicry (VM)

## Abstract

Cadherins mediate cohesive contacts between isotypic cells by homophilic interaction and prevent contact between heterotypic cells. Breast cancer cells neighboring endothelial cells (ECs) atypically express vascular endothelial (VE)-cadherin. To understand this EC-induced VE-cadherin expression in breast cancer cells, MCF7 and MDA-MB-231 cells expressing different endogenous cadherins were co-cultured with ECs and analyzed for VE-cadherin at the transcriptional level and by confocal microscopy, flow cytometry, and immunoblotting. After losing their endogenous cadherins and neo-expression of VE-cadherin, these cells integrated into an EC monolayer without compromising the barrier function instantly. However, they induced the death of nearby ECs. EC-derived extracellular vesicles (EVs) contained soluble and membrane-anchored forms of VE-cadherin. Only the latter was re-utilized by the cancer cells. In a reporter gene assay, EC-adjacent cancer cells also showed a juxtacrine but no paracrine activation of the endogenous VE-cadherin gene. This cadherin switch enabled intimate contact between cancer and endothelial cells in a chicken chorioallantoic membrane tumor model showing vasculogenic mimicry (VM). This EV-mediated, EC-induced cadherin switch in breast cancer cells and the neo-expression of VE-cadherin mechanistically explain the mutual communication in the tumor microenvironment. Hence, it may be a target to tackle VM, which is often found in breast cancers of poor prognosis.

## 1. Introduction

Cadherins are a family of cell adhesion molecules that enable cells to seclude themselves from cells of other tissues, as well as to interact and communicate with the same kind of cells. The extracellular regions of cadherins are responsible for homotypic binding to the ectodomains of other cadherin molecules of the same isoform, which are presented on neighboring cells [1]. Additionally, they may interact with other receptors. For example, vascular endothelial cadherin (VE-cadherin) can associate with vascular endothelial growth factor (VEGF) receptor II (VEGFRII, also known as Flk1 or KDR) to reduce its proliferative signaling [2].

Intracellularly, the cadherin cytoplasmic tails interact with several proteins, such as p120, and α- and β-catenins, thereby connecting intercellular contacts with the actin cytoskeleton and cell-signaling pathways [3]. All classical cadherins share similar structures, comprising five extracellular cadherin repeats, a transmembrane (TM) domain, and an intracellular domain (ICD). They can be sub-divided into type I cadherin that have a histidine–alanine–valine (HAV) motif present in the first EC domain, and type II cadherins without such a HAV motif. While E- and N-cadherin are type I cadherins, VE-cadherin is a type II cadherin [4]. E- and N-cadherin only differ in the catenin isoforms that bind to their ICDs—E-cadherin binds with the shorter isoform of p120 catenin while N-cadherin interacts with the longer isoform [5]. Of great importance in cancer pathophysiology, changes in these two-cell surface cadherins, including switches between the cadherin types, occur during epithelial-to-mesenchymal transition (EMT), thereby altering cell migration and tumor invasiveness. EMT is an initial step of metastatic expansion, in which tumor cells can acquire stem cell phenotypes and become resistant to cancer therapy [6,7,8]. During EMT, loss, decrease, or dysfunction of E-cadherin is consistently observed in most of the advanced, undifferentiated, and aggressive carcinomas of the mammary gland and other epithelial tissues [9,10]. Consequently, β-catenin is released from the cancer cell membrane and, after its translocation into the nucleus, regulates transcription of several genes. For example, the β-catenin/TCF pathway activates the vimentin promoter, resulting in epithelial cell migration and tumor cell dissemination and invasion [11]. Re-expression of E-cadherin in these cancer cells reverts EMT [12,13]. Hence, E-cadherin–based cell interaction is an essential factor in tumor invasiveness [14,15]. However, in some tumors, decreased expression of E-cadherin does not necessarily correlate with increased cancer cell motility or invasion [16]. Instead, N-cadherin may also promote an invasive phenotype in breast cancer cells despite their high E-cadherin expression [16].

VE-cadherin, initially described to be typical for intercellular junctions between endothelial cells (ECs) [17], can also be expressed by cancer cells, as detected in human invasive breast carcinoma sections [18]. There, VE-cadherin is found at three subcellular sites: in the cytoplasm, at the cell membrane, and in the nucleus. Forced expression of VE-cadherin in breast cancer cells induced collective MDA-MB-231 breast cancer cell migration and promoted their integration into endothelial monolayer as well as the formation of functionally competent cell junctions. VE-cadherin expression reportedly increases during EMT in v-Ha-Ras-transformed mammary epithelial cells and enhances tumor growth in vivo [19]. However, very little is known about the mechanisms of increased VE-cadherin expression in the cancer cells and about the impact it may have on interactions between ECs and tumor cells (TCs), especially in breast cancer cells.

Orchestrating TC survival and progression, the tumor microenvironment (TME) is characterized by the biochemical composition and biophysical properties of the extracellular matrix (ECM) by ECM-sequestered cytokines, as well as by the mutual interactions of the different cellular components, such as TCs, cancer-associated fibroblasts, immune cells, and ECs [20,21]. Within the TME, all these cells communicate through several juxtacrine and paracrine mechanisms. The paracrine mechanisms include communication via soluble factors, such as cytokine and extracellular vesicles (EVs). Cells form EVs by outward budding of the plasma membrane or by an intracellular endocytic trafficking pathway involving the fusion of multivesicular late endocytic compartments with the plasma membrane [22,23]. Exosomes, a subclass of EVs, are extracellular nanovesicles with a typical diameter of 50–150 nm, being characterized by different membrane proteins, such as CD63. Secreted from cancer cells, they can carry nucleic acids, lipids, and proteins [24]. The uptake of exosomes by the acceptor cells can happen through phagocytosis, a type of endocytosis [25].

As a consequence, the exosome-transported cargos are released intracellularly into the acceptor cell and may actively influence its phenotype [26]. Thus, other cells of the TME cells acquire a prometastatic phenotype and support tumor functions such as tumor angiogenesis [27]. Moreover, other types of TME cells, such as ECs, secrete exosomes with EC-typical cargos, which may influence TCs to acquire EC-like characteristics such as formation of vasculogenic mimicry vessels [28,29]. Aggressive tumors possess mosaic and vasculogenic mimicry vessels, which are lined by both ECs and TCs, of which the latter integrate into the endothelium and replace ECs partially or entirely, respectively [20,30,31].

To understand the molecular mechanism in terms of how tumor cells can form close intercellular contacts with ECs, we hypothesized that juxtacrine or paracrine signaling between ECs and TCs could induce ectopic VE-cadherin expression in cancer cells and thereby support the molecular mimicry of TCs to contact neighboring ECs. The present work provides novel insights into the mutual communication and exchange of material between TCs and ECs, which enables breast cancer cells to express endothelial-specific markers such as VE-cadherin. Aggressive and non-aggressive breast cancer cells show neo-expression of VE-cadherin in a mechanism that depends on EVs released by ECs. This highlights a novel type of mutual interaction between these two cell types within the TME. In contrast to the type II cadherin (cadherin-11), the expression of type I cadherin (E-cadherin) inhibits the integration of TCs into the EC layer. VE-cadherin expression in non-aggressive TCs decreases E-cadherin exposure on the cell surface. Thus, the ratio of VE-cadherin to E-cadherin on the cell surface likely determines the integration of the TCs into the EC layer and allows better cohesion with ECs in 3D tumor spheroids. Therefore, the expression of VE-cadherin in non-invasive cancer cells promotes cancer progression to a more aggressive and invasive phenotype.

## 2. Results

### 2.1. Expression of VE-Cadherin, VEGFRI, and VEGFRII Is Induced in Breast Cancer Cells during Co-Culture with Endothelial Cells

**Figure 1:**
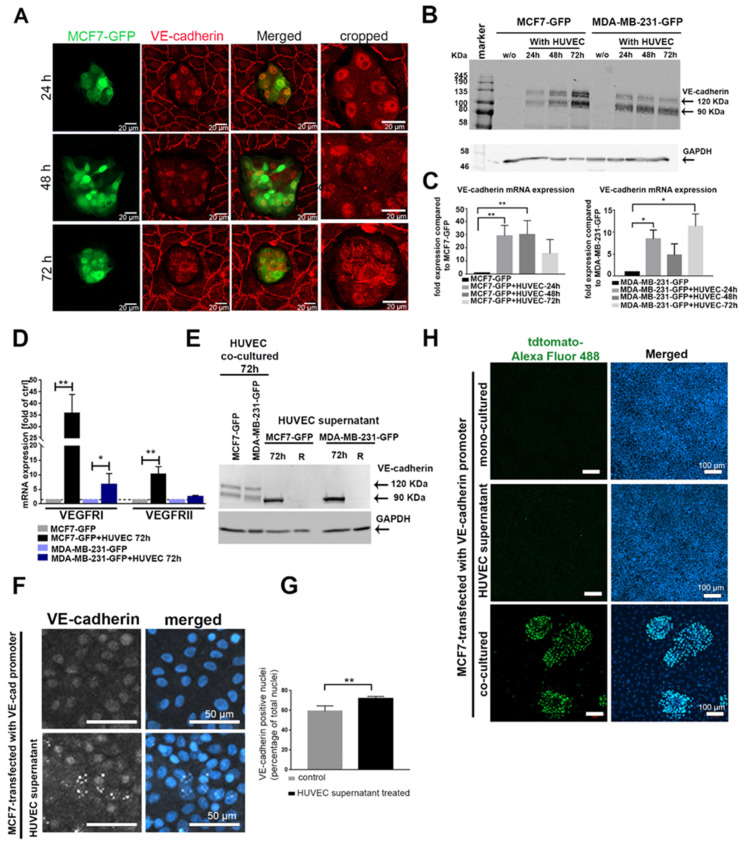
Cancer–endothelial cell interaction induces expression of endothelial cell (EC) markers in breast cancer cells. (**A**) Immunofluorescent staining for vascular endothelial cadherin (VE-cadherin) (red) in MCF7-green fluorescent protein (GFP) cells (green), which were added onto a human umbilical vein endothelial cell (HUVEC) monolayer for 24, 48, and 72 h. The protein (**B**) (full western blot figures (Figure S8) and mRNA (**C**) levels of VE-cadherin were analyzed in GFP-labeled cancer cells, MCF7-GFP and MDA-MB-231-GFP, which were isolated by fluorescence-activated cell sorting (FACS) after co-culturing with HUVECs for different time points (24, 48, and 72 h). Values are presented as means ± SD (*n* = 3) (* *p* ≤ 0.05; ** *p* ≤ 0.01). (**D**) The mRNA levels of vascular endothelial growth factor receptor I (VEGFRI), and VEGFRII were determined by qPCR in the isolated MCF7-GFP and MDA-MB-231-GFP cells. Values are presented as means ± SD of the fold changes as compared to the monocultured tumor cells (TCs) (*n* = 3) (* *p* ≤ 0.05; ** *p* ≤ 0.01) (**E**) The soluble VE-cadherin ectodomains, soluble VE (sVE)-cadherin, shedded by HUVECs into the cell supernatant, were detected in cancer cell lysates with the BV9 antibody by Western blot. sVE-cadherin was not stable in cancer cells and was lost within 24 h (lane R) after the removal of the HUVEC-conditioned medium (full western blot figure. As a positive control, lysates of cancer cells co-cultured with HUVECs were used (two left lanes). (**F**) Immunofluorescence labeling of VE-cadherin in MCF7 cells treated with HUVEC medium for 48 h showed increased VE-cadherin-positive signal in the nucleus. (**G**) The positive VE-cadherin staining in the nucleus was biometrically quantified by ImageJ. For the calculation of VE-cadherin-positive signal in the nucleus, we evaluated *n* = 141 Ctrl cells (gray bar), and *n* = 130 MCF7-cells treated with HUVEC supernatant (black bar) for 24 h. Means values ± SD are shown (** *p* ≤ 0.01). (**H**) MCF7 cells transfected with the VE-cadherin-tdTomato reporter gene were treated with HUVEC culture supernatant, co-cultured with HUVECs (positive control), or monocultured (negative control). The activity of VE-cadherin promoter was quantified by staining the cells with a primary antibody against tdTomato and secondary antibody against tdTomato conjugated with Alexa Fluor 488 (green). Western blots of (**B**,**E**) are shown Figure S8, (**G**) is shown in Figure S9, (**C**) is shown in Figure S10.

Tumor progression goes along with altered cell–cell contacts with respect to both homotypic and heterotypic interactions. Cancer cells may lose their homotypic interactions during dissemination and interact with other cell types. Intercellular contacts are predominantly mediated via cadherins, which homotypically allow communications between the same cell types. Therefore, we examined the cadherin repertoire of breast cancer cells, which were co-cultured in direct cellular contacts with human umbilical vein endothelial cells (HUVECs). To this end, we first characterized the expression of different cadherins (E-cadherin, N-cadherin, cadherin-11, and VE-cadherin) in a panel of breast cancer cell lines with different degrees of aggressiveness (Figure S1A). By using vimentin as a marker for TC aggressiveness, we selected two non-aggressive, E-cadherin-expressing cell lines (MCF7 and T47D) and two aggressive, E-cadherin-negative cell lines (MDA-MB-231 and BT549) for our study (Figure S1B). None of these four cancer cell lines expressed VE-cadherin (Figure S1C). To study the impact of heterotypic cell interactions on cadherin expression of cancer cells, we added green fluorescent protein (GFP)-expressing breast cancer cells, MCF7-GFP or MDA-MB-231-GFP, onto the apical surface of an EC monolayer. MCF7-GFP cells were monitored for the expression and localization of VE-cadherin over different time points (24, 48, and 72 h) by immunofluorescence (Figure 1A). In parallel, we analyzed the expression of VE-cadherin at the protein and mRNA levels in the GFP-positive cancer cells isolated from the co-culture system by fluorescence-activated cell sorting (FACS) (Figure 1B,C). VE-cadherin, typically found in ECs, was detected ectopically in cancer cell clusters (Figure 1A). Its expression was detected at the translational and transcriptional levels in MCF7 and MDA-MB-231 cells after 24 h of co-culture, remaining above the VE-cadherin levels of monocultured TC controls at 48 h and 72 h (Figure 1B,C). Fluorescence microscopy showed that VE-cadherin was enriched and localized at the cell membrane after 72 h of co-culturing (Figure 1A). In addition, other endothelial cell markers such as VEGFRI and VEGFRII were expressed at the transcriptional level in MCF7 and MDA-MB-231 after co-culturing with HUVECs (Figure 1D).

To analyze whether ECs could also induce VE-cadherin expression in breast cancer cells in a paracrine manner, independently of direct cell–cell contact, we treated MCF7 and MDA-MB-231 with HUVEC supernatant (collected after 72 h culture), and tested their VE-cadherin content and changes after 24, 48, and 72 h by Western blots (Figure 1E and Figure S2A). However, only an approximately 90 kDa large VE-cadherin fragment, instead of the full-length VE-cadherin (120 kDa) was detected. It was found in the supernatant of monocultured HUVECs (Figure S2B,C). To pinpoint which domains of VE-cadherin this fragment contains, we repeated the immunoblots with epitope-specific antibodies. An antibody directed against the VE-cadherin extracellular domain (BV9) detected the ≈90 kDa band of the HUVEC supernatant (Figure S2B), whereas an antibody directed against the intracellular domain (C-19) did not (Figure S2C). Thus, only the 90 kDa soluble VE-cadherin ectodomains, termed sVE-cadherin, but not the full length-VE-cadherin, was detectable within the cancer cells. It is likely shed from the HUVECs, and TCs take up this sVE-cadherin released by HUVECs. However, the sVE-cadherin does not persist within the cancer cells for long, as after replacing the HUVEC-conditioned medium (CM), the sVE-cadherin band vanished within 24 h (lane labeled R in Figure 1E). To localize its intracellular localization, we used immunofluorescence. The signal of the uptaken sVE-cadherin was prominently found in the cell nucleus (Figure 1F,G). Hence, sVE-cadherin from the HUVEC supernatant failed to be correctly localized to the intercellular contact sites of TCs, likely due to its lack of the transmembrane domain. Furthermore, the HUVEC-conditioned medium with its sVE-cadherin was insufficient to induce VE-cadherin expression in TCs, as MCF7 cells that had been transfected with VE-cadherin-tdTomato reporter gene failed to activate VE-cadherin promoter in response to HUVEC-conditioned medium. In contrast, the VE-cadherin gene promoter was activated in the same MCF cells when grown in a mixed co-culture with HUVECs (Figure 1H).

### 2.2. EC-Induced VE-Cadherin Expression Also Occurred in Breast Cancer Cells In Vivo

**Figure 2:**
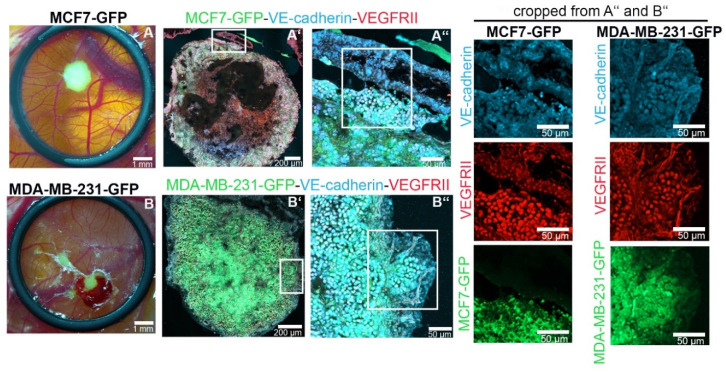
VE-cadherin expression is induced in TCs in the vicinity of endothelial cells (ECs) in vivo. MCF7-GFP (green, in (**A**,**A’**,**A’’**)) and MDA-MB-231-GFP (green in (**B**,**B’**,**B’’**)) cells formed tumors after 7 days of inoculation in a chorioallantoic membrane (CAM) implantation assay. Immunofluorescence staining of tumor cryosections with VE-cadherin (light blue) and VEGFRII (red) is shown. The CAM tissue is seen on the top right corner of the MCF7 and MDA-MB-231 sections in (**A’’**,**B’’**), respectively.

To analyze whether ectopic expression of VE-cadherin also occurs in tumors formed by MCF7 and MDA-MB-231 in vivo, we xeotransplanted cancer cells into the chorioallantoic membrane (CAM) of chicken embryos. Tolerated by chicken embryos due to their immature immune system, human cancer cells grew on the surface of the CAM for 6 days (Figure 2). The GFP-tagged tumor cells expressed VE-cadherin. The signal of VE-cadherin-positive cells prevailed at the invasive tumor front and at the periphery of tumor islands, where infiltrating ECs line up into tube structures (Figure 2).

### 2.3. EC-Induced VE-Cadherin Expression in Tumor Cells Resulted in Downregulation of Endogenous Cadherins and Dislocation of β-Catenin from Cell Junctions

**Figure 3:**
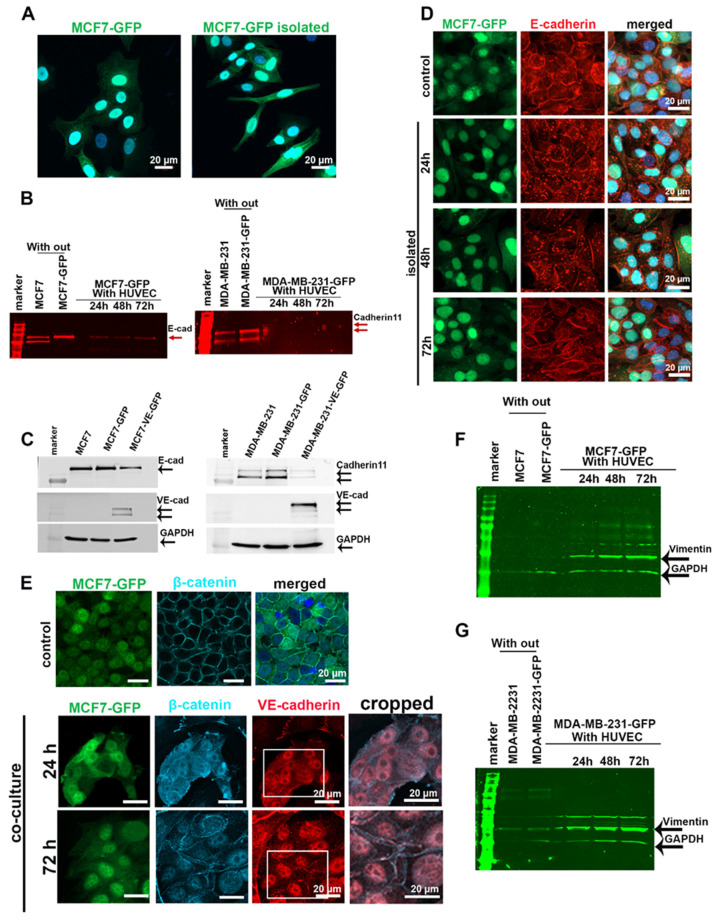
Endogenous cadherins in MCF7 and MDA-MB-231 cells were downregulated after co-culturing with HUVECs. (**A**) After having been co-cultured with HUVECs for 5 days, MCF7-GFP cells exhibited an elongated cell shape, resembling a more mesenchymal phenotype. (**B**) MCF7-GFP and MDA-MB-231-GFP cells co-cultured with HUVECs over different time points (24, 48, and 72 h) were isolated by FACS and lysed to quantify the expression level of E-cadherin in MCF7 and cadherin-11 in MDA-MB-231 cells by Western blotting by using a secondary antibody with a detection signal in the 700 nm channel of the Li-Cor Odyssey Infrared Reading System. Bands are shown in red. The GAPDH loading controls are shown in (**F**,**G**), which are the same Western blot membranes. (**C**) Forced expression of VE-cadherin-GFP in MCF7 and MDA-MB-231 cells induced downregulation of endogenous cadherin. Monocultured MCF7 and MDA-MB-231 cells, either non-transduced or transduced to express VE-cadherin, were lysed and analyzed for their endogenous cadherins. An equal concentration of samples (50 µg) were loaded onto polyacrylamide gels. (**D**) After having been co-cultured with HUVECs for 5 days, MCF7-GFP cells were isolated by FACS and further monocultured for 24, 48, and 72 h. Even 48 h after cancer cell isolation, immunofluorescent staining showed that E-cadherin (red) is internalized and degraded (red). (**E**) Immunofluorescent staining of β-catenin in control (MCF7-GFP monoculture) and in MCF7-GFP cells that were co-cultured with HUVECs revealed an overall but transient displacement of β-catenin from intercellular cell contacts. (**F**,**G**) Western blot analysis of vimentin expression levels in MCF7-GFP cells (**F**) and MDA-MB-231-GFP cells (**G**) after isolation from co-culture showed that MCF7 cells, and to a less extent also MDA-231 cells, increased their vimentin expression during coculture with HUVECs. To this end, the same Western blot membranes of (**B**) were stained for vimentin and GAPDH using corresponding secondary antibodies with an emission signal in the 800 nm channel of Li-Cor Odyssey Infrared Reading System. The bands are hued in green. Western blots of (**B**,**C**,**F**) are shown in Figure S9.

After 5 days of co-culture with HUVECs, more than 50% of the MCF7-GFP cells changed their morphology from an epithelial to an elongated appearance, similar to cells undergoing epithelial–mesenchymal transition (EMT) (Figure 3A). To test whether EC-induced morphological changes and ectopic VE-cadherin expression could affect the expression of the endogenous cadherins, we co-cultured MCF7-GFP or MDA-MB-231-GFP cells with HUVECs for 24, 48, and 72 h. The fluorescent cancer cells were isolated by FACS, lysed, and analyzed for their E-cadherin and cadherin-11 protein and mRNA levels. In contrast to the gradual increase of VE-cadherin expression in cancer cells, the protein amounts of E-cadherin in MCF7 and cadherin-11 in MDA-MB-231 cells decreased significantly after 24 h of co-culturing (Figure 3B). However, the mRNA level of endogenous cadherins did not decrease, and instead markedly increased after 24 h of co-culturing and decreased thereafter (Figure S3A,B). This suggested that the expression of endogenous cadherins in tumor cells is regulated at the protein level in response to contact with ECs. To determine whether increased VE-cadherin expression in TCs is responsible for the downregulation of E-cadherin and cadherin-11, we transduced breast cancer cell lines with a VE-cadherin–GFP-encoding lentiviral vector. Forced expression of VE-cadherin repressed the endogenous cadherins in MCF7 and MDA-MB-231 cells, irrespective of their co-culture with ECs (Figure 3C). Albeit to a lesser extent, the endogenous cadherins were also downregulated in cancer cells without any forced VE-cadherin expression, but were co-cultured with HUVECs.

To understand the fate of E-cadherin in MCF7-GFP cells after co-culture with HUVECs, we sorted the GFP-tagged cancer cells from the co-culture and analyzed E-cadherin localization by immunofluorescence microscopy. E-cadherin downregulation goes along with the dislocation of this protein from the cell–cell junctions and its accumulation in enlarged vesicles inside the cytoplasm of the MCF7 cell (Figure 3D). This dislocation in EC-co-cultured MCF7 cells was transient and reversible. It did not persist after the TCs were separated from ECs, as 72 h after cell separation, isolated TCs re-exposed E-cadherin at cell–cell contacts (Figure 3D). E-cadherin/β-catenin complexes maintain the integrity of epithelial cell–cell contact and prevent Wnt/β-catenin signaling [32]. E-cadherin downregulation in MCF7 cells co-cultured with HUVECs is followed by nuclear accumulation of β-catenin in these TCs during the first 24 h of co-culturing. β-Catenin partially relocalized at cell–cell contacts along the plasma membrane after 72 h of co-culture. This happened concomitantly with the induction of VE-cadherin in MCF7 cells, and at some sites, both proteins co-localized (Figure 3E). Similarly, vimentin expression was progressively upregulated in MCF7 and MDA-MB-231 cell co-cultures with ECs (Figure 3F,G). Together with increased vimentin expression, these data suggested that the Wnt/β-catenin signaling pathway is activated during the EC-induced cadherin switch from E- to VE-cadherin in MCF7 cells and from cadherin-11 to VE-cadherin in MDA-MB231 cells.

### 2.4. VE-Cadherin Expression in Breast Cancer Cells Promoted their Association with ECs and Integration into Endothelial Tubes

**Figure 4:**
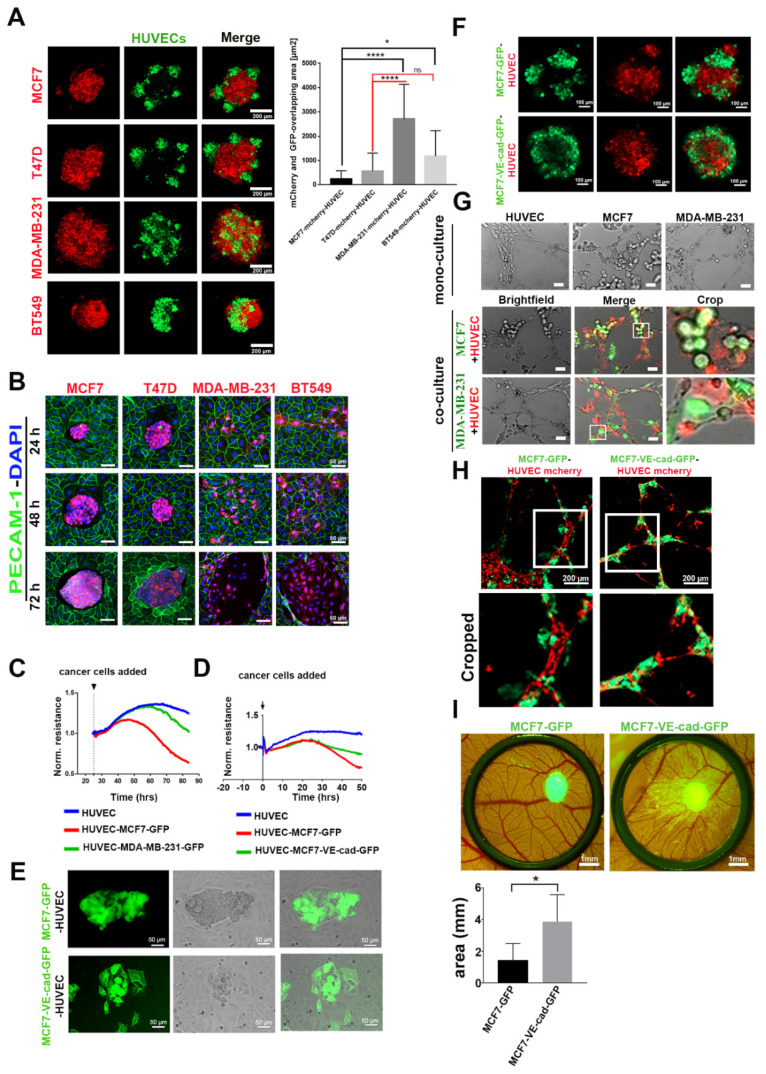
Expression of VE-cadherin in MCF7 cells promoted the integration of cancer cells into the HUVEC monolayer and endothelial tube-like structures. (**A**) Three-dimensional co-culture of HUVEC-GFP cells with E-cadherin-expressing cancer cell lines (MCF7-mCherry and T47D-mCherry) and E-cadherin-negative cell lines (MDA-MB-231-mCherry and BT549-mCherry). The graph shows the quantification of the colocalized regions between cancer cells and HUVECs in spheroids, which were measured by using the CellProfiler software. E-cadherin-containing cancer cells intermingled with HUVECs less than cancer cells expressing other cadherins. For the evaluation of overlapping area, the numbers of images analyzed were as follows: *n* = 17 MCF7-mCherry, *n* = 14 T47D-mCherry, *n* = 13 MDA-MB-231-mCherry, and *n* = 14 BT549-mCherry. Mean values ± SD are shown (* *p* ≤ 0.05; **** *p* ≤ 0.0001) (**B**) In 2D co-culture, E-cadherin-positive cell lines (MCF7-mCherry and T47D-mCherry) and E-cadherin-negative cells (MDA-MB-231-mCherry and BT549-mCherry) were added to the HUVEC monolayer, and the interaction was monitored over different time points (24, 48, and 72 h). (**C**) Transendothelial electrical resistance (TER) measurement of HUVEC monolayer showing normalized resistance (averaged resistance of the entire period) without or with co-culturing with MCF7-GFP and MDA-MB-231-GFP cells. The numbers of samples analyzed were as follows: *n* = 4 HUVEC, *n* = 6 HUVEC MCF7-GFP, and *n* = 6 HUVEC-MDA-MB-231. (**D**) Impedance spectroscopy measurements of a HUVEC monolayer without or with co-culturing with MCF7, either expressing GFP or a VE-cadherin-GFP-fusion protein. The numbers of samples analyzed were as follows: *n* = 4 HUVEC, *n* = 6 HUVEC-MCF7-GFP, and *n* = 6 HUVEC-MCF7-VE-cad-GFP. Among the latter two, VE-cadherin-GFP expression showed higher TER values. (**E**) The MCF7-VE-cadherin-GFP interacted individually with the HUVEC monolayer in the 2D co-culture system. (**F**) Heterospheroids of MCF7-GFP or MCF7-VE-cadherin-GFP mixed with HUVEC-mCherry for 24 h. (**G**) MCF7 and MDA-MB-231 cells (green) seeded alone or together with HUVECs (red) on Matrigel for the tube formation assay. (**H**) MCF7-VE-cadherin-GFP integrated better in the tube-like structure as compared to MCF7-GFP cells. (**I**) CAM invasion by MCF7-GFP or MCF7-VE-cadherin-GFP cells on day 14. Biometric evaluation included eight images from three chicken embryos per cell line from three independent experiments. The area occupied by metastatic foci was calculated by ImageJ software. Means values ± SD are shown (* *p* ≤ 0.05).

To characterize the function of EC-induced VE-cadherin expression in breast cancer cells, we analyzed TC–EC interaction of both invasive and non-invasive breast cancer cells, employing different approaches. In a 3D heterospheroid model consisting of cancer cells (red) and HUVECs (green), E-cadherin-negative cell lines (MDA-MB-231 and BT549) intermingled with ECs more prominently than E-cadherin-positive cell lines (MCF7 and T47D) (Figure 4A). In a 2D culture system, cancer cells were added onto a monolayer of confluent HUVECs. In this experiment, E-cadherin-positive cells did not interact with ECs individually, but remained in clusters on top of the EC monolayer (Figure 4B). They proliferated in these homotypic aggregates until they reached a specific spheroid size at about 48 h and then seemed to oust the surrounding HUVEC layer (Figure 4B). In contrast, E-cadherin-negative cells interacted individually with HUVECs but did not show any visible signs of endothelial monolayer disintegration until after 72 h of co-culturing, when they broke up the HUVEC layer (Figure 4B).

The integrity of the endothelial monolayer was monitored in real time by transendothelial electrical resistance (TER) measurement. E-cadherin-positive MCF7-GFP caused a significant decrease of TER of the EC monolayer after 48 h, whereas E-cadherin-negative MDA-MB-231 cells did not reduce TER until 72 h, when the EC monolayer disintegrated (Figure 4C). The integration of individual MDA-MB-231 cells did not compromise the barrier function of the EC monolayer. Next, we examined how the forced expression of VE-cadherin expression in cancer cells, identifiable by a GFP-tagged VE-cadherin, would influence the interplay between TCs and ECs. After 37 h of adding cancer cells into the impedance spectroscopy measurement, MCF7-VE-cadherin-GFP cells did not decrease the TER value as much as MCF7-GFP cells (Figure 4D). In line with TER measurement results, individual MCF7-VE-cadherin-GFP cells intermingled with ECs more than the GFP-expressing MCF7 cells, similar to the invasive breast cancer cell lines MDA-MB-231 and BT549 (Figure 4E). In the 3D model, MCF7-VE-cadherin-GFP cells (green) cohered better with ECs and formed a more homogenous spheroid compared to MCF7-GFP cells (Figure 4F).

To examine the integration of VE-cadherin-expressing cancer cells into endothelial tube structures, we included MCF7 and MDA-MB-231 in a tube formation assay with fluorescently labeled HUVECs in a Matrigel system. In monoculture, only HUVECs formed capillary-like tubular structures on Matrigel after 3 h (Figure 4G). In contrast, when MCF7 or MDA-MB-231 were seeded together with HUVECs, they integrated into the ECs cords. The invasive MDA-MB-231 cells integrated better than MCF7 cells (Figure 4G). Forced expression of VE-cadherin in MCF7 cells significantly increased their ability to incorporate into HUVEC tubular structures when compared to MCF7-GFP cells (Figure 4H). Eventually, in the CAM assay, MCF7-VE-cadherin-GFP cells invaded more than MCF7-GFP cells, similar to the in vitro assays (Figure 4I). Thus, neo-expression of VE-cadherin improved the cell-to-cell contact between TCs and ECs in non-invasive breast cancer cells.

### 2.5. Extracellular Vesicles (EVs) Were Involved in the Mechanism of EC-Induced VE-Cadherin Expression in Breast Cancer Cells

**Figure 5:**
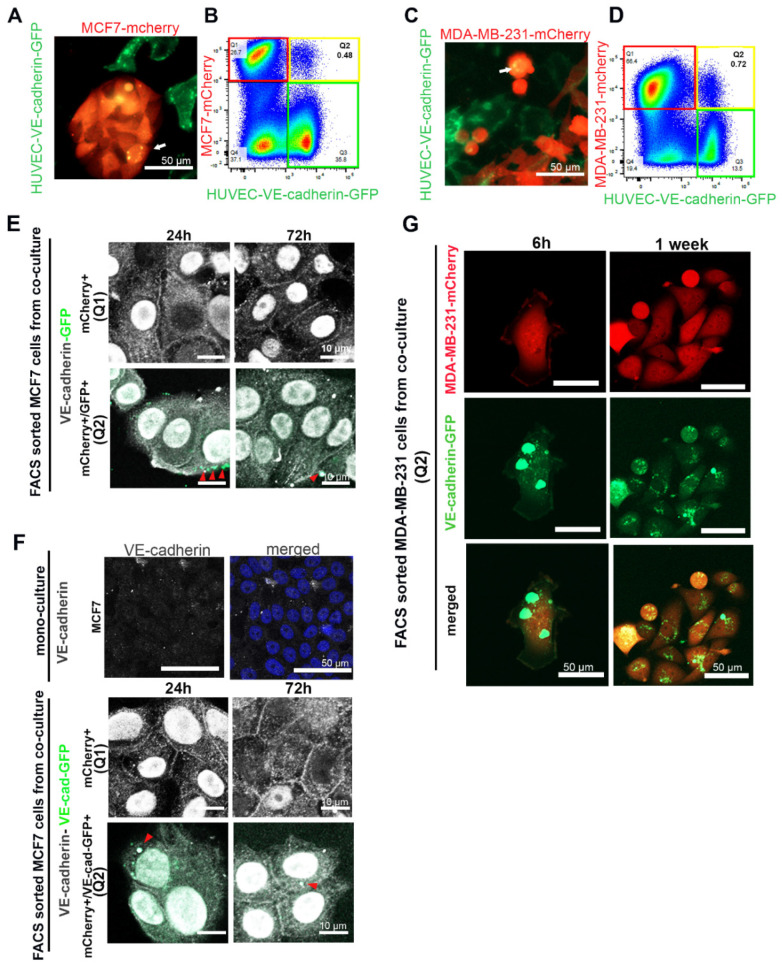
Cancer cells took up VE-cadherin from HUVECs. (**A**) MCF7-mCherry or (**C**) MDA-MB-231-mCherry were co-cultured with HUVECs expressing VE-cadherin-GFP. The arrows show VE-cadherin-GFP-containing vesicles that were taken up in breast cancer cells. (**B**) MCF7-mCherry or (**D**) MDA-MB-231-mCherry co-cultured with HUVEC-VE-cadherin-GFP for 72 h were analyzed by flow cytometry. Red frames denote the mCherry-positive cell population Q1 (MCF7 or MDA-MB-231 cells), green frames indicate VE-cadherin-GFP-expressing HUVECs, and yellow frames highlight the population Q2 of mCherry+/GFP+ double-positive cells. (**E**,**F**) After MCF7-mCherry cells were co-cultured for 3 days with (**E**) HUVECs-GFP or (**F**) HUVECs-VE-cadherin-GFP, we isolated mCherry+ single-positive cells (Q1) and mCherry+/GFP+ double-positive cells (Q2) and stained them with an antibody against VE-cadherin (C-19) (shown in gray). VE-cadherin localized at cell–cell junctions and in the nucleus of isolated cells. Arrows indicate GFP-positive (**E**) or VE-cadherin-GFP containing (**F**) EC-derived membrane vesicles taken up by MCF7-mCherry+/GFP+ cells. These vesicles were also positively stained for VE-cadherin by immunofluorescence staining (gray intensity). MCF7 cell monoculture was used as a negative control for VE-cadherin staining. (**G**) After having been co-cultured with VE-cadherin-GFP-expressing HUVECs for 72 h, MDA-MB-231-mCherry cells were isolated with FACS and cultured for 1 week in the absence of HUVECs. Even 1 week after isolation, VE-cadherin-GFP vesicles could be detected in the MDA-MB-231 cells.

VE-cadherin expression is tissue-specifically assigned to ECs. To understand the molecular mechanism of EC-induced VE-cadherin expression in breast cancer cells, we introduced the VE-cadherin-GFP fusion construct into HUVECs by lentiviral transduction. After co-culturing mCherry-labelled TCs, MCF7 and MDA-MB-231, with HUVEC-expressing VE-cadherin-GFP, we observed the presence of EC-produced VE-cadherin-GFP in breast cancer cells by live-cell imaging and flow cytometry (Figure 5A–D). After 72 h of co-culturing, VE-cadherin-GFP signals were detected in breast cancer cells by confocal microscopy (yellow spots in Figure 5A,C).

Flow cytometry analyses revealed a small population Q2 (in Figure 5B,D) of cells (<1%) that were positive for both mCherry and GFP, markers for cancer cells and VE-cadherin-GFP, respectively, while a larger portion of cancer cells, population Q1 (in Figure 5B,D), did not contain GFP signals. As the GFP tag is fused to the carboxyl terminus of full-length VE-cadherin, we hypothesized that GFP-tagged VE-cadherin is transferred from ECs to TCs via membrane vesicles, which after budding include EC cytoplasm. Therefore, we also transduced HUVECs with GFP-encoding lentivector, which led to GFP expression in the cytoplasm of ECs. When these GFP-labeled HUVECs were co-cultured with MCF7-mCherry, T47D-mCherry, MDA-MB-231-mCherry, or BT549-mCherry cells for 72 h, all four breast cancer cell lines contained fractions of cells stained double-positive for mCherry and GFP in flow cytometry (Figure S4A). Therefore, cancer cells took up membrane-anchored VE-cadherin from neighboring HUVECs via extracellular membrane vesicles (EVs). This transfer was time-dependent and increased over the incubation time up to 72 h (Figure S4B).

To confirm whether VE-cadherin was transported by EVs from ECs to the TCs, we co-cultured mCherry-labelled MCF7 cells with HUVECs, which expressed either GFP (Figure 5E) or VE-cadherin-GFP (Figure 5F). The FACS-sorted mCherry+ cells (Figure 5E,F panels Q1) and mCherry+/GFP+ (double-positive) cells (Figure 5E,F panels Q2) were allowed to adhere and were stained with an antibody against the cytoplasmic domain of VE-cadherin (C-19). This staining revealed the total amount of VE-cadherin expressed by the cancer cells, both the endogenous VE-cadherin that was synthesized during TC–EC interaction (Figure 5E,F, panels Q1) and the GFP-tagged VE-cadherin transferred from neighboring ECs via EVs (Figure 5E,F, panels Q2). In both cell populations, Q1 and Q2, the total VE-cadherin similarly localized at the cell–cell junctions, at cytoplasmic vesicles, and in the nuclei over 72 h after isolation (Figure 5E,F). Likely due to its low amount, VE-cadherin-GFP-positive vesicles (green) could only be detected after the mCherry-positive MCF7 (Figure 5F (Q2) or MDA-MB-231 (Figure 5G) cells co-cultured with VE-cadherin-GFP-expressing HUVECs were sorted for both mCherry+/GFP+ (double-positive cells). However, the EC-derived GFP-tagged VE-cadherin was localized weakly at cell–cell junctions, but prominently in cytoplasmic vesicles. During the following EC-free cultivation of these cells, the vesicles diminished in size, with little restoration of a VE-cadherin signal at the intercellular junctions (Figure 5G).

**Figure 6:**
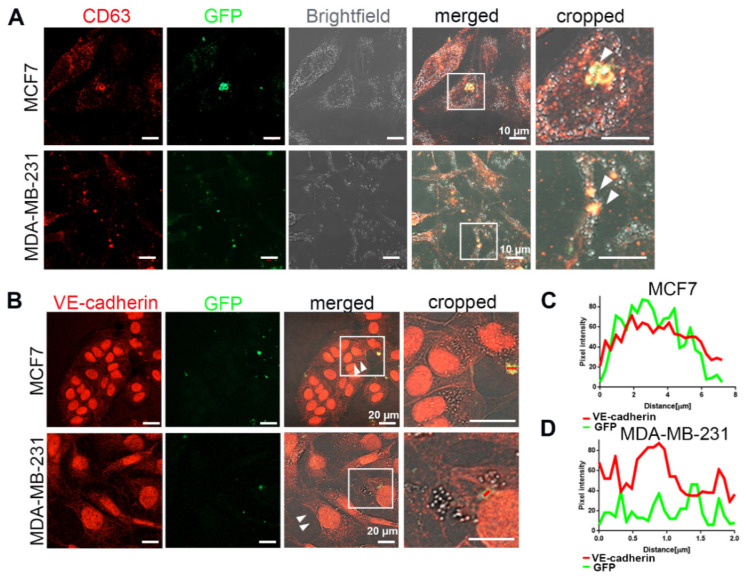
VE-cadherin was transferred from ECs to cancer cells via EVs. (**A**) After 6 days of co-culturing MCF7-mCherry or MDA-MB-231-mCherry with GFP-labeled HUVECs, the extracellular vesicles were isolated from conditioned medium with Exo-spin column and added to MCF7 or MDA-MB-231 monoculture. White arrows showing CD63-GFP vesicles inside the breast cancer cells indicate the exosome transfer from HUVEC-GFP to cancer cells. (**B**) Incubation of MCF7 (upper panel) or MDA-MB-231 (lower panel) cells for 5 days with the EVs isolated from co-culture supernatant induced VE-cadherin (red) expression and its recruitment at the junctions (arrows) in breast cancer cells. (**C**,**D**) Line scan (red bars) from images of (**B**) quantifying the presence of VE-cadherin and GFP signal in the EVs derived from GFP-labeled HUVECs after being internalized by (**C**) MCF7 and (**D**) MDA-MB-231 cells.

To understand whether HUVEC-derived EVs transfer functionally active VE-cadherin to TCs, we isolated EVs from the conditioned medium of GFP-labelled HUVECs co-cultured with mCherry-labelled breast cancer cells, MCF7 or MDA-MB-231 cells, and added them to the naive TCs. Six days after this treatment, abundant EVs of different sizes, containing GFP; VE-cadherin; and CD63, a characteristic marker for EVs [33], were observed in the cytoplasm of MCF7 and MDA-MB-231 (Figure 6). Noteworthy, upon treatment with EVs, we found some weak staining of VE-cadherin at cell–cell contacts (arrows) in MCF7 and MDA-MB-231 (Figure 6B). These data demonstrated that EVs released from nearby HUVECs were taken up by breast cancer cells, thereby transferring EC-derived VE-cadherin protein or VE-cadherin-encoding mRNA to TCs. To test whether VE-cadherin could be detected on EVs from HUVECs, we isolated EVs from HUVEC supernatant. They carried different contents depending on whether the HUVECs were in monoculture or co-culture with the breast cancer cells (Figure S5). The full-length of VE-cadherin protein (120 KDa) could only be detected in the EVs isolated from co-culture supernatant, but not in the monoculture HUVEC supernatant, whereas the 90 kDa VE-cadherin fragment was found in both conditions. These results suggest that cancer cells influence the contents of EVs secreted by HUVECs into the culture supernatant, and that only the membrane-anchored full-length VE-cadherin activates VE-cadherin gene expression in breast cancer cells.

### 2.6. Cancer Cells Phagocytosed Extracellular Vesicles from ECs

**Figure 7:**
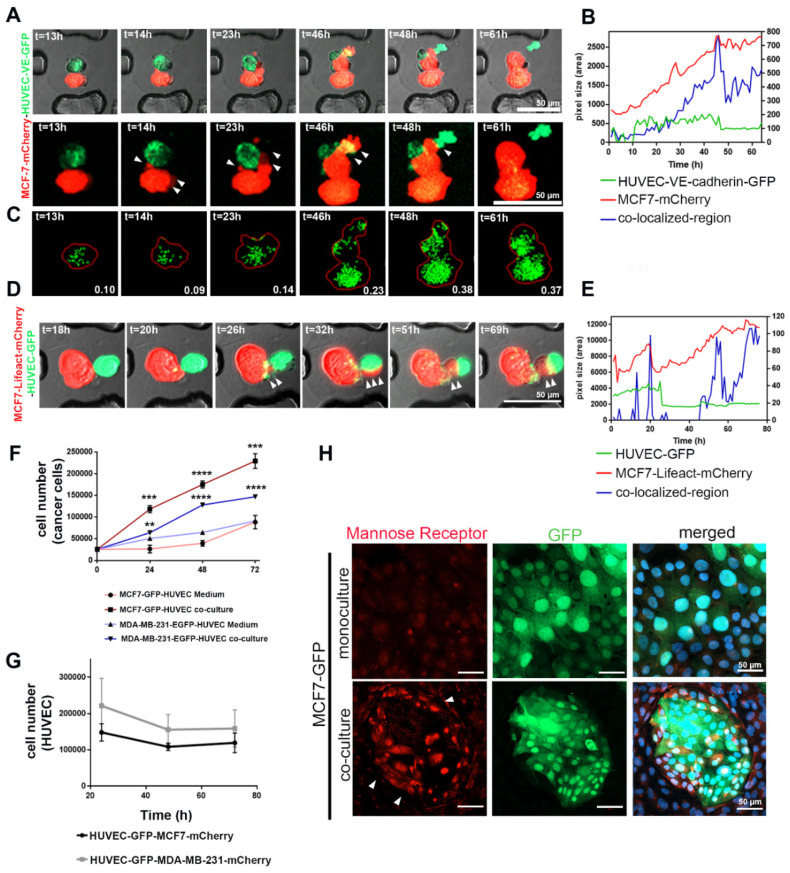
Phagocytosis contributed to the internalization of HUVEC-secreted EVs by cancer cells, as well as the opposite mutual effects between the two cell types on proliferation. (**A**) The MCF7-mCherry and HUVEC-expressing VE-cadherin-GFP or (**D**) MCF7 expressing lifeact-mCherry and HUVEC-GFP were encapsulated inside agarose beads and monitored by live-cell imaging. Images were taken with confocal microscopy and 10× magnification at 37 °C. (**B**,**E**) The fluorescence area of mCherry (MCF7 cells), GFP, or VE-cadherin-GFP (HUVECs), as well as the area of colocalized fluorescence, were quantified from time-lapse microscopy images ((**A**,**D**) respectively) by using the CellProfiler software. (**C**) The CellProfiler software visualized VE-cadherin-GFP vesicles transferred from HUVECs to MCF7-mCherry, enabling their quantification. (**F**) Time course of proliferation of MCF7 and MDA-MB-231 cell lines during co-culture with HUVECs or treated with HUVEC-conditioned medium for different time points (24, 48, and 72 h). Values are presented as means ± SD (*n* = 3) (** *p* ≤ 0.01; *** *p* ≤ 0.001; **** *p* ≤ 0.0001). (**G**) The proliferation of HUVECs co-cultured with TCs for 24, 48, and 72 h was analyzed by using flow cytometry (*n* = 4). (**H**) MCF7-GFP cells in culture without and with HUVECs for 72 h were immunofluorescently stained for mannose receptor (red). Its expression was involved in efferocytosis, especially in the TCs, which were close to HUVECs (arrows).

To study the mechanism of how cancer cells internalize HUVEC-derived EVs, we investigated the interaction of MCF7-mCherry with VE-cadherin-GFP- or cytoplasmic GFP-expressing (Figure 7) HUVECs in 3D by using novel cell-on-chip (EVORION) technology. First, the TCs and ECs were encapsulated within agarose beads, which were then immobilized within a microchip cell array. Live-cell imaging allowed us to monitor the interaction between the cells. The fluorescent areas of mCherry (MCF7 cells), GFP (HUVEC-expressing VE-cadherin-GFP or cytoplasmic GFP) and of both fluorophores in colocalization were quantified time-dependently by using the CellProfiler software (Figure 7B,E). In contrast to the mCherry area, which increased gradually, the area of the GFP signal did not change significantly over time (Figure 7B,E), indicating that TCs and ECs grow differently within the encapsulated beads.

To follow up on the mutual pro- and antiproliferative effects of HUVECs and cancer cells, we co-cultured mCherry-labeled cancer cells, MCF7 and MDA-MB-231, with HUVEC-GFP in a 2D co-culture system and analyzed cell proliferation by flow cytometry. Similar to our 3D culture results, flow cytometry showed a significant increase in the number of TCs, whereas the number of HUVEC-GFP cells decreased over-time (Figure 7F,G and Figure S6A). The proliferative effect of HUVECs on cancer cells requires direct interaction between the two cell types since the addition of HUVEC-conditioned medium to TCs only poorly supported their proliferation (Figure 7F). In contrast, TCs exerted an antiproliferative effect on HUVECs (Figure 7G). Whereas the addition of collected TC-conditioned medium (CM) from MCF7 and MDA-MB-231 weakly decreased proliferation of HUVECs (Figure S6B), the CM of the TC–EC co-culture significantly reduced viability and numbers of HUVECs (Figure S6B). Thus, in co-culture, TCs induced EC death, however, without typical signs of apoptosis, such as annexin V staining (Figure S6C).

Morphologically, MCF7 cancer cells form lamellipodia after 32–42 h of co-culturing (Figure 7A). mCherry-tagged lifeact proved cytoskeleton remodeling in MCF7 cells (Figure 7D). Lamellipodia formation and cytoskeleton reorganization would support the hypothesis that phagocytic clearance of dying cells (efferocytosis) is a mechanism by which MCF7 cells take up extracellular vesicles from dying HUVECs (Figure 7A,D) [34,35]. Therefore, the area of colocalized mCherry and GFP fluorescence increased over time (Figure 7B,E) due to the increasing number of HUVEC-derived, GFP-containing vesicles inside TCs (Figure 7C). Upregulation of the mannose receptor within the ingesting TCs additionally points to efferocytosis [36]. Its expression is increased in the TC–EC co-culture system, especially at sites where the GFP-labelled MCF7 cells neighbor HUVECs (Figure 7H).

## 3. Discussion

In aggressive tumor tissues, VE-cadherin is localized in the cytoplasm of all VE-cadherin-expressing TCs, with additional nuclear and cell membrane localization in some cases [18]. However, the process mediating this neo-expression has not been elucidated. Our present work demonstrates that VE-cadherin is expressed in breast cancer cells neighboring ECs. Moreover, it unravels possible biological mechanisms of this EC-induced VE-cadherin expression in TCs. The cadherin molecules at adheren junctions are diverse, some of which influence growth factor receptor signaling and Rho GTPases to promote cell motility and invasion [37]. E-cadherin is preferentially expressed in cells of epithelial origin, while VE-cadherin is specific to ECs. VE-cadherin regulates the intercellular contacts between ECs, thereby determining the integrity of blood vessels and barrier function [17].

Here, we demonstrated that (i) HUVECs release VE-cadherin-containing EVs, the composition of which changes if cancer cells are close by. Moreover, we showed that (ii) breast cancer cells take up EC-produced VE-cadherin molecules of distinct lengths, which undergo different fates within the cancer cells. Furthermore, (iii) cancer cells carry out efferocytosis towards neighboring ECs, which, albeit supportive to cancer cell proliferation, undergo necroptosis. (iv) Via different mechanisms, EC induces VE-cadherin expression in breast cancer cells, which is accompanied by a decrease of originally expressed cadherin type. As a consequence, (v) this cadherin switch, especially the increase of VE-cadherin in combination with a loss of E-cadherin, enables breast cancer cells to integrate into endothelial tubes without significant loss of vessel integrity and barrier function. The full-length VE-cadherin in cancer cells, both endogenously produced and transferred from ECs via EVs, localized within intracellular vesicles and at intercellular junctions.

In our studies, two phenotypically different types of breast cancer cells (MCF7 and MDA-MB-231) were employed in co-culture models with HUVEC cells. While the MCF7 breast cancer cell line was derived from an invasive ductal carcinoma patient sample and represented a luminal A breast cancer subtype, MDA-MB-231 was isolated from a breast cancer adenocarcinoma, triple-negative subtype [38]. When co-cultured with ECs, ectopic expression of VE-cadherin enriched at cell–cell junctions can be observed in both MCF7 and MDA-MB-231 for at least 1 week after isolation. Competition between cadherins for their clustering at intercellular junctions in the same cell was shown in ECs between VE-cadherin and N-cadherin as a regulatory mechanism for modulating cadherin function and signaling [39]. In TCs, VE-cadherin presents structural features that are responsible for its ability to exclude E-cadherin and cadherin-11 from cell–cell contacts in both MCF7 and MDA-MB-231, respectively, as well as to induce their internalization and downregulation. However, in a 3D culture model, ECs induced EMT in breast cancer cells and were able to switch from E-cadherin to N-cadherin expression [40]. Therefore, we cannot rule out the fact that factors other than the increase of VE-cadherin expression might contribute to the decrease of E-cadherin expression in the TCs that neighbor ECs.

Furthermore, E-cadherin downregulation led to β-catenin dislocation from MCF7 cell–cell contacts. Conversely, the expression of VE-cadherin was progressively upregulated. As the increase of VE-cadherin lagged behind the decrease of E-cadherin, it is conceivable that β-catenin transiently disappeared from the intracellular junction and reappeared there only 72 h after co-culturing. This goes in line with the cadherin switch described by other researchers [37]. For instance, such a cadherin switch was described during EMT, in which E-cadherin disappeared and N-cadherin appeared in epithelial cancers, and together with morphological changes, they increased expression of the mesenchymal marker vimentin and enhanced cell motility. Furthermore, downregulation of the originally expressed cadherin type led to carcinoma cell disaggregation from their homotypic neighboring cells. Degradation of endogenous cadherin was considered as a consequence of competition for binding to p120-catenin [41].

The effect of VE-cadherin neo-expression seems to be more relevant for the E-cadherin-expressing cell line MCF7, as compared to MDA-MB-231. E-cadherin belongs to type I classical cadherins while cadherin-11 and VE-cadherin belong to type II classical cadherins. In vitro cell aggregation assays showed that type II cadherins mediate both homophilic adhesive interactions between cells expressing identical cadherins and selective heterophilic interactions between cells expressing different cadherins [42]. Therefore, our data suggest that in MCF7 cells, not only the acquisition of VE-cadherin, but also the decrease of E-cadherin plays a vital role in diminishing the homotypic cell–cell cohesion and switching to a heterotypic cell–cell interaction. VE-cadherin expression has been observed in specific cancer types, including aggressive melanoma associated with vasculogenic mimicry and with trans-differentiation and stem-like phenotype [43]. VE-cadherin expression in MCF7 cells leads to a change in phenotype, both morphologically and functionally. MCF7 co-culture with ECs leads to morphologic changes towards an elongated cell shape, transient displacement of β-catenin from the cell–cell junction, an increase of vimentin expression, an increase of VE-cadherin expression, and a concomitant decrease in E-cadherin expression. This cadherin switch may contribute to vascular mimicry as it enables TCs to interact with ECs and to integrate into the endothelial monolayer and consequently increase TC invasion, as confirmed by the CAM assay.

Our data reveal the potential mechanisms of how TCs acquire VE-cadherin expression. We discovered that VE-cadherin could be transferred from ECs via EVs to cancer cells. EVs contain different cytosolic and membrane proteins derived from the parent cell. They can transfer functional proteins, nucleic acids, and lipids between cells in vitro and in vivo, and therefore are capable of changing the composition and function of recipient cells. Exosomes are the smallest subset of EVs, with a size ranging from 30–150 nm, and apoptotic bodies are the largest of all EVs (up to 5000 nm) and are released as membrane blebs of cells undergoing apoptosis [44]. By labeling HUVECs with GFP-tagged VE-cadherin or GFP, we showed that EVs of different sizes originate from HUVECs and are incorporated into MCF7 and MDA-MB-231 cells. EVs generated by ECs could induce the expression of VE-cadherin in breast cancer cells. In target cells, EVs can influence the physiology of cells by transcription of competent RNA molecules [45]. Moreover, EVs can transport proteins related to essential signaling pathways such as mitogen-activated protein kinase (MAPK), nuclear factor κB (NFκB), and protein kinase B (AKT) to the recipient cells. These proteins and miRNAs play essential roles in processes associated with cell proliferation [46].

Among the mutual interactions between TCs and ECs, we pinpointed a second VE-cadherin transfer mechanism, in which material transfer from ECs occurs due to the influence of TCs on EC viability, as highlighted in our co-culture experiments, and previously suggested by others [47]. Our results demonstrated that TCs cause programmed necrosis (necroptosis) [47] of ECs and that debris of HUVECs are then incorporated by TCs via efferocytosis, thereby enabling the TCs to obtain EC-typical proteins such as VE-cadherin. Prior to efferocytosis, TCs that are in contact with ECs upregulate the mannose receptor, which is a crucial receptor that enables TCs to endocytose cell debris and material. For this reason, the mannose receptor is a potential cell maker for this process and could be used to identify TCs capable of performing efferocytosis and, as a consequence, receive EC-secreted VE-cadherin-containing material.

Lastly, we could identify a third mechanism by which TCs receive VE-cadherin, which is not the transmembrane full-length form of VE-cadherin, but the soluble VE-cadherin ectodomain with its characteristic molecular mass of 90 kDa. This VE-cadherin side product is a consequence of shedding by enzymes such as ADAM10, as previously shown [48]. The generation of sVE-cadherin was associated with inflammation-induced breakdown of endothelial barrier functions [48]. Interestingly, the presence of full-length VE-cadherin could only be found in the supernatant of the co-culture and the lysate of HUVECs, whereas sVE-cadherin was also detected in the supernatant of monocultured HUVECs. Obviously, the TCs can influence the composition of EVs, which are released by the ECs. Interestingly, only the co-culture supernatant with the TC-induced EVs of HUVECs promoted the expression of the VE-cadherin gene in MCF7 cells. While full-length VE-cadherin could be detected in TC lysates for at least 1 week, the sVE-cadherin was transiently detected and was lost after 24 h of treatment with HUVEC supernatant. Mechanistically, sVE-cadherin does not seem to influence TCs directly but can destabilize the ECs monolayer and indirectly contribute to a more invasive TC phenotype [48].

Although it is not clear if these different mechanisms of EC-induced VE-cadherin expression in TCs happen simultaneously or sequentially, the release of full-length VE-cadherin depends on juxtacrine cell communication between ECs and TCs. If not direct cell–cell contacts, at least a close proximity of ECs and TCs are required for the EC-induced VE-cadherin expression and the subsequent cadherin switch in TCs. These conditions are met in the TME.

## 4. Materials and Methods

### 4.1. Cell Culture

Breast carcinoma cell lines (MCF7, T47D, HCC1806, HCC1937, BT549, MDA-MB-453, SUM149, MDA-MB-468, SKBR3, BT20, and BT549) were kindly provided by Dr. M. Götte (Department of Gynecology and Obstetrics, Münster, Germany) and Dr. B. Greve (Department of Radiation Oncology, Münster, Germany). Cells were maintained in DMEM/high glucose medium (Lonza, Basel, Switzerland) (SKBr3, MDA-MB-453, and MDA-MB-468) or in RPMI medium (MCF7, T47D, HCC1806, HCC1937, BT20, and BT549). Both media were supplemented with 10% fetal calf serum (FCS; Gibco, Waltham, MA, USA) and 100 U/mL penicillin–streptomycin (PS; Gibco). SUM149 cells were cultured in Dulbecco’s modified Eagle’s medium-F12 (1:1) with 5% FCS, insulin (5 μg/mL) (Merk, Darmstadt, Germany), and hydrocortisone (1 μg/mL; Qiagen, Hilden, Germany). MDA-MB-231 cell line was obtained from DSMZ (Leibniz Institute DSMZ–German Collection of Microorganisms and Cell Cultures) and was grown in DMEM/high glucose medium containing 10% FCS and 100 U/mL PS. HUVECs were kindly provided by Dr. D. Vestweber (Max Planck Institute of Molecular Biomedicine, Münster, Germany) or purchased from Promocell (Heidelberg, Germany) and cultured up to passage five in ECGM-2 medium supplemented with SupplementPack (PromoCell).

### 4.2. PiggyBac Transposon-Based Reporter Expression in MCF7 Cells

MCF7 cells were trypsinized and centrifuged at 200× *g* for 10 min. One million cells were resuspended in 100 μL Nucleofector Kit V reagent (Lonza, Basel, Switzerland). Then, the cells were mixed with 0.5 μg transposase plasmid and 5 μg transposon plasmid containing VE-cadherin promoter fragments from positions −3394 to +39 followed by tdTomato, kindly provided by Dr. I. Slukvin (Department of Pathology and Laboratory Medicine, Madison, WI, USA), and were then electroporated using program Q-001 according to the manufacturer’s protocol (Amaxa, Cologne, Germany). The electroporated cells were resuspended with RPMI growth medium in a 6-well plate. Selection with 100 μg/mL zeocin (Invitrogen, Toulouse, France) began 3 days after electroporation.

### 4.3. Lentiviral Transduction of Target Cells and Generation of Stable Cell Lines

Viral particles were produced by transient co-transfection of 293T cells with lentivirus encoded in the psPAX2 plasmid, the envelope elements from the SVS (somatitis virus) encoded in the pMD2.G plasmid, and the vector genome encoded in the pLV-CMV-MCS-SV40-Puro and pLenti-puro transfer plasmids, with GeneJammer transfection reagents (Agilent, Waldbronn, Germany). Conditioned medium containing lentivirus was harvested 72 h after transfection, cleared by low-speed centrifugation, filtered through 0.45 μm pore-size cellulose acetate filters, and concentrated with Lenti-X concentrator (Clontech, Saint-Germain-en-Laye, France). For transduction, cells were seeded in a 6-well plate and incubated overnight in 2 mL culture medium. Concentrated virus particles in 100 μL suspension with 10 μg/mL polybrene were diluted in 500 μL ECGM-2 medium, added to the cells, and incubated for 24 h. After 48 h, cells were selected with 1 μg/mL puromycin (Toku-E, Sint-Denijs-Westrem, Belgium). Transduction efficacy was assessed by flow cytometry analysis. Plasmids psPAX2 and pMD2.G were kindly provided by Dr. D. Trono (Lausanne, Switzerland). Transfer plasmids (pLenti-EGFP, pLenti-mCherry, and pLenti-LifeAct-mCherry) were generous gifts from Dr. S. Huveneers (AMC, Amsterdam, The Netherlands), and pLVX-IRES-Puro-human VE-cadherin-EGFP was provided by Dr. D. Vestweber (Max Planck Institute of Molecular Biomedicine, Münster, Germany).

### 4.4. Analysis of Cell–Cell Interaction at Single-Cell Resolution in a 3D Environment

MCF7 cells (MCF7-mCherry or MCF7-lifeact-mCherry cell lines) and HUVEC-GFP cells were co-encapsulated in hydrogel beads as described previously [49,50]. Briefly, for the co-encapsulation, 75 μL of HUVEC cell suspension (16,000 cells μL^−1^) and 75 μL of MCF7 cell suspension (13,000 cells μL^−1^) were mixed in phosphate-buffered saline (PBS). Cell suspension (150 μL) was then mixed with 150 μL 3% (w/v) SeaPrep agarose (Lonza). Agarose droplets containing cells were generated in a bead formation chip by using syringe pumps (EVORION Biotechnologies, Münster, Germany). After agarose droplet gelation, beads containing cells were transferred and immobilized into a trapping chip by using a control unit (EVORION Biotechnologies). After immobilization of hydrogel beads, the trapping chip was perfused with medium (ECGM-2 and RPMI mixture (50:50) supplemented with 20% FCS and 20 mM HEPES). TC–EC interaction was monitored by time-lapse confocal microscopy (LSM800) at 37 °C with 10× magnification (Figure S7). Images were taken with 1 h time intervals over a total of 61–68 h. Images were analyzed with the open-source software CellProfiler (www.cellprofiler.org). A Cell Profiler pipeline consisting of several individual imaging analysis modules was constructed to identify and quantify objects in every image automatically. The pipeline first segments the images into objects (MCF7-mCherry or MCF7-lifeact-mCherry cells—red channel, and HUVEC-GFP cells—green channel) and then makes a comparison between the objects in each channel to measure colocalization. The detected MCF7 cells were marked as the region of the interest, and the area of GFP pixel (HUVEC-GFP), as well as the number of green particles, were calculated within the region of interest to measure the HUVEC-derived extracellular vesicles taken up by MCF7 cells.

### 4.5. Homo- and Heterospheroid Formation

Spheroids were formed as previously described [51]. The cancer cells used in this assay—MCF7-mCherry, MDA-MB-231-mCherry, T47D-mCherry, BT549-mCherry, MCF7-GFP, and MCF7-VE-GFP—were co-cultured with HUVEC-GFP cells of passage 2–3. Each spheroid contained 5000 cells. Cells were resuspended in a solution composed of one-quarter volume of a 6 mg/mL methylcellulose (Sigma-Aldrich, Deisenhofen, Germany) solution and three-quarter volume of cell culture media. This solution was distributed in a round-bottom 96-well plate, 100 μL per well, and incubated at 37 °C for 24 h. For heterospheroid formation, cancer cells were mixed in a 1:2 ratio with HUVECs. Images were acquired using a confocal microscope (LSM 700, Zeiss, Oberkochen, Germany) after 24, 48, and 72 h.

### 4.6. Chick Chorioallantoic Membrane (CAM) Assay with MCF7 and MDA-MB-231 Cells

Freshly fertilized chicken eggs were purchased from Brinkschulte GmbH (Senden, Germany). Eggs were incubated for 72 h at >60% relative humidity and 37 °C. On developmental day 3, the eggs were cracked open, and the embryo was carefully transferred into a plastic square weighing boat (89 × 89 × 25 mm). The weighing boat was placed in a round transparent glass Petri dish of 100 × 20 mm, and 40 mL of purified water was added to ensure sufficient humidity. On day 10, 10^6^ cancer cells (MCF7-GFP, MCF7-VE-GFP, or MDA-MB-231-GFP) were embedded in collagen gel, as described previously [52]. A Teflon ring was placed on the CAM membrane, and the embedded cancer cells were grafted into the ring. The images of the region containing the disc on the CAM were taken with the stereomicroscope (Nikon, Tokyo, Japan). The tumors were dissected on day 17 of chick development. Tumors were fixed in 4% formaldehyde for 15 min, washed thrice in PBS, and transferred into 10% sucrose for 3 h at 4 °C and 30% sucrose overnight at 4 °C. Tumors were then embedded in tissue-freezing medium and cut with a cryotome (Leica Biosystems, Leica Biosystems, Wetzlar, Germany) into 8 μm thick sections. The experiments were performed according to the guidelines of the European Parliament (2010/63/EU) and the council for the protection of animals in research (§14 TierSchVersV).

### 4.7. Immunofluorescence Staining of Cells and Frozen Tumor Sections

Cells (30 × 10^3^) were seeded on 8-well chamber ibiTreat Surface slides (ibidi, Martinsried, Germany), and incubated at 37 °C and 5% CO_2_. Cells were fixed with freshly prepared 2% formaldehyde dissolved in PBS, and 24 h later were washed with PBS for 10 min, then permeabilized with 0.1% Triton X-100 for 10 min at 4 °C. Cells were stained with the primary antibody diluted in blocking buffer (2% horse serum and 1% bovine serum albumin in PBS) at 4 °C, overnight. After washing three times, cells were incubated with Alexa Fluor-conjugated secondary antibodies (1:200; Invitrogen, Karlsruhe, Germany) for 1 h at room temperature. Nuclei were stained with 200 ng/mL DAPI (4′-6-diamidino-2-phenylindole; Sigma, Deisenhofen, Germany) in PBS. The cells were finally washed twice in PBS and mounted in a fluorescent mounting medium (Dako, Hamburg, Germany). Pictures were acquired using a confocal microscope LSM800 with an oil immersion 40× objective (Zeiss, Oberkochen, Germany). For fluorescent staining of frozen tissue, frozen 8 μm sections were rehydrated in PBS, pH 7.3, for 5 min, and stained as described above.

The primary antibodies were goat anti-human VE-cadherin (1:50; Santa Cruz Biotechnology, Inc., Dallas, TX, USA), mouse anti-cadherin 11 (1:50; Invitrogen. Eugene, OR, USA), mouse anti-E-cadherin (1:100; BD Biosciences, San Jose, CA, USA), mouse anti-β-catenin (1:100; BD Biosciences), rabbit anti-N-cadherin (1:50; Abcam, Cambridge, MA, USA), mouse anti-vimentin (1:50; BD Biosciences), rabbit anti-mannose receptor antibody (1:50; Abcam), rat anti-NRP1 Ab (1:20; Pineda, Berlin, Germany), mouse Anti-CD63 (1:50; Abcam), and rabbit anti-VEGF receptor II (1:20; Abcam).

### 4.8. Immunoblot Analysis

An equal number of cells was plated in 6 cm dishes and cultivated for 48 h. The cells were first rinsed with cold PBS. Then, 250 μL of lysis buffer (10mM Tris, 1mM EDTA, 150mM NaCl, 0.5% NP-40) supplemented with Complete protease inhibitor cocktail (Roche, Basel, Switzerland) were added to the cells and incubated on ice for 5 min. Cells were scraped off on ice, and cell lysates were collected. The protein concentration was determined with the BCA Protein Assay Kit (Thermo Fisher Scientific, Waltham, MA, USA). Lysate proteins were separated by SDS-PAGE in a 10% polyacrylamide gel and transferred by wet blotting onto nitrocellulose membranes (Whatman, Dassel, Germany). Membranes were incubated with the following primary antibodies: goat anti-VE-cadherin (C-19) (1:100; Santa Cruz, Dallas, TX, USA), mouse anti-VE-cadherin (BV9) (1:100; Santa Cruz), mouse anti-Cadherin 11 (1:50; Invitrogen), mouse anti-E-cadherin (1:100; BD Biosciences), or mouse anti-vimentin (1:50; BD Biosciences), followed by incubation with HRP-conjugated secondary IgG (1:1000; Dako, Hamburg, Germany).

For detection of immunoblots with fluorescently labelled antibodies, we incubated membranes with IRDye 600 CW and 800 CW infrared secondary anti-mouse- and anti-rabbit-IgG antibodies (1:10,000, Li-COR Biotechnology, Bad Homburg, Germany). Immunoreactive bands were detected with the Li-COR Infrared Reading System according to the manufacturer’s instructions (Li-Cor Odyssey Infrared Reading System, Homburg, Germany).

### 4.9. RNA Isolation and Reverse Transcription PCR Analysis

Total RNA was isolated from cell lysates using the RNeasy Mini Kit (Qiagen, Hilden, Germany) and was reverse transcribed using a Reverse Transcriptase Kit (Qiagen). Real-time PCR reactions were performed with a RotorGene SYBR Green PCR Kit in the RotorGene Cycler (both Qiagen). The following primers were used: E-cadherin-fw, 5′- GACCGGTGCAATCTTCAAA -3′ and E-cadherin-rev, 5′-TTGACGCCGAGAGCTACAC-3′; cadherin-11-fw, 5′-TTGGTCACTCAACAAATGACAA-3′ and cadherin-11-rev, 5′-GTTGCGTCCACCCTCAAG-3′; VE-cadherin-fw, 5′-CATCTTCCCAGGAGGAACAG-3′ and VE-cadherin-rev, 5′-AGAGCTCCACTCACGCTCAG-3′; VEGFRI-fw, 5′-TTTGCCTGAAATGGTGAGTAAGG-3′ and VEGFRI -rev, 5′-TGGTTTGCTTGAGCTGTGTTC -3′; VEGFRII-fw, 5′-GGCCCAATAATCAGAGTGGCA-3′ and VEGFRII-rev, 5′-CCAGTGTCATTTCCGATCACTTT-3′; NRP1-fw, 5′-TTGCAGTCTCTGTCCTCCAA 3′ and NRP1-rev, 5′-GAAAAATGCGAATGGCTGAT-3′; and TOP1-fw, 5′-CCAGACGGAAGCTCGGAAAC-3′ and TOP1-rev, 5′-GTCCAGGAGGCTCTATCTTGAA -3′. Cycle threshold (Ct) values were normalized by the ∆∆Ct method [53], and TOP1 was used as a housekeeping gene.

### 4.10. Electric Cell-Substrate Impedance Sensing

HUVECs were seeded onto L-cysteine-reduced, fibronectin-coated 8W10E electrodes (Applied Biophysics, Troy, NY, USA). Electrical impedance was measured at 4000 Hz in real time at 37 °C and 5% CO_2_ using the ECIS ΖΘ system (Applied Biophysics, Troy, NY, USA). After 24 h, 10 × 10^3^ cancer cells (MCF7 or MDA-MB-231) were added to each well on the confluent HUVEC layer for 72 h.

### 4.11. Endothelial Tube Formation

This assay was performed as described previously [54]. In a μ-Slide angiogenesis chamber (ibidi), 10 μL of Matrigel were solidified at 37 °C within 30–60 min. On this Matrigel, 5 × 10^3^ HUVECs were cultured at 37 °C in ECGM-2 medium (PromoCell, Heidelberg, Germany), and tube formation was recorded for 6–18 h with images acquired every 10 min (IncuCyte ZOOM, Essen BioScience, Welwyn Garden City, United Kingdom). To set up the tumor–endothelial cell tube formation, we mixed cancer cells (MCF7 or MDA-MB-231) with HUVECs in a 1:1 ratio and seeded them onto Matrigel-coated ibdi chambers.

### 4.12. Flow Cytometry

After co-culturing, cells were harvested with accutase according to the manufacturer’s instructions (Chemicon, Millipore, Darmstadt, Germany). Cell sorting and analysis were performed on a FACSAria IIIu cell sorter (BD Biosciences) using an 85 µm nozzle. Strict forward scatter pulse height vs. pulse width gating was used to exclude cell doublets from the analysis. Flow cytometric data were analyzed using FlowJo software (BD Biosciences).

### 4.13. Apoptosis Assays

After the TCs (MCF7-mCherry or MDA-MB-231-mCherry) were co-cultured with HUVEC-GFP cells over different time points (24, 48, and 72 h), we harvested cells with accutase according to the manufacturer’s instructions (Chemicon, Millipore, Darmstadt, Germany), centrifuged them at 250× *g* for 4 min, and resuspended them in the 1× annexin V binding buffer (BD Biosciences). Cells were stained with Alexa Fluor 647-labeled annexin V (1:20, Biolegend, San Diego, CA, USA) and 1.43 μM DAPI to distinguish apoptotic from necrotic cells. All samples were analyzed on a FACSAria IIIu cell sorter (BD Biosciences). Data analysis was performed using FlowJo software (BD Biosciences).

### 4.14. Exosome Isolation from Cell Culture Medium (CCM)

Exo-spin precipitation was carried out according to the manufacturer’s instructions (Cell Guidance Systems, Cambridge, United Kingdom). Briefly, 10 mL cell culture medium was collected and centrifuged at 300× *g* for 10 min to remove cells. The supernatant was then transferred to a new centrifuge tube and spun at 16,000× *g* for 30 min to remove any remaining cell debris. Exo-spin buffer at a 2:1 ratio was mixed with clarified CCM and incubated overnight at 4 °C. The sample was then spun at 20,000× *g* for 30 min, the supernatant was discarded, and the pellet was resuspended in 100 µL of PBS. The sample was further purified using the provided columns, and exosomes were eluted in 200 µL of PBS.

### 4.15. Statistics

Statistical analyses were performed using GraphPad Prism 6 software (GraphPad Software, La Jolla, CA, USA). Comparisons between two groups were conducted with Student’s *t*-test, and analyses among more than two groups were performed using one-way ANOVA followed by Tukey’s post hoc test. Differences with *p* values < 0.05 were considered significant.

## 5. Conclusions

In conclusion, our data highlight the communication between ECs and TCs within the TME. The communication is mediated inter alia via EC-derived extracellular vesicles, which support the TCs to undergo a cadherin switch, as they change their original cadherin type and express VE-cadherin. This molecular mimicry enables the TCs to get in close contact with the endothelium of ingrowing tumor vessels. Moreover, it may explain the integration of TCs into vasculogenic mimicry vessels, which characterize tumors with poorer prognosis. Hence, understanding this process may help to prevent the formation of vasculogenic mimicry vessels. Preventing the EC-induced cadherin switch in TCs might be a relevant strategy to curb tumor progression in patients.

## Supplementary Materials

The following are available online at https://www.mdpi.com/2072-6694/12/8/2138/s1. Figure S1: Characterization of different breast cancer cell lines; Figure S2: The soluble VE-cadherin ectodomain, termedsVE-cadherin,wasdetected in cancer cells treated with HUVEC supernatant; Figure S3: mRNA levels of endogenous cadherin changed in MCF7 and MDA-MB-231 cells after co-culturing with HUVECs; Figure S4: GFP labeled EVs of HUVECs are transferred from ECs to TCs; Figure S5: TCs influence the contents of EVs secreted by HUVECs; Figure S6: Cancer cells induce cell death in HUVECs, but HUVECs promote cancer cell proliferation via direct cell-cell interaction; Figure S7: Scheme of cell-cell interaction at single-cell resolution in 3D environment. HUVEC and MCF7 cell suspensions were mixed in PBS. Figure S8: Western blots of Figure 1B,E. Figure S9: Western blots of Figure 3B,C,F and Figure 1G. Figure S10: Western blots of Figure 1C. Figure S11: Western blots of Figure S2. Figure S12: Western blots of Figure S5.

## Abbreviations

CAM	chick chorioallantoic membrane
GFP	green fluorescent protein
HUVECs	human umbilical vein endothelial cells
ECs	endothelial cells
ECM	extracellular matrix
EMT	epithelial-to-mesenchymal transition
EVs	extracellular vesicles
FACS	fluorescence-activated cell sorting
sVE-cadherin	soluble VE-cadherin
TCs	tumor cells
TER	transendothelial electrical resistance
TME	tumor microenvironment
VE-cadherin	vascular endothelial cadherin
VEGFR	vascular endothelial growth factor receptor

## References

[1] Leckband D., Prakasam A. (2006). Mechanism and dynamics of cadherin adhesion. Annu. Rev. Biomed. Eng..

[2] Dejana E., Orsenigo F., Lampugnani M.G. (2008). The role of adherens junctions and VE-cadherin in the control of vascular permeability. J. Cell Sci..

[3] Yap A.S., Kovacs E.M. (2003). Direct cadherin-activated cell signaling: A view from the plasma membrane. J. Cell Biol..

[4] Basu R., Taylor M.R., Williams M.E. (2015). The classic cadherins in synaptic specificity. Cell Adh. Migr..

[5] Loh C.Y., Chai J.Y., Tang T.F., Wong W.F., Sethi G., Shanmugam M.K., Chong P.P., Looi C.Y. (2019). The E-Cadherin and N-Cadherin Switch in Epithelial-to-Mesenchymal Transition: Signaling, Therapeutic Implications, and Challenges. Cells.

[6] Cardiff R.D. (2005). Epithelial to Mesenchymal Transition Tumors: Fallacious or Snail’s Pace?. Clin. Cancer Res..

[7] Nieto M.A., Huang R.Y., Jackson R.A., Thiery J.P. (2016). EMT: 2016. Cell.

[8] Zhang P., Sun Y., Ma L. (2015). ZEB1: At the crossroads of epithelial-mesenchymal transition, metastasis and therapy resistance. Cell Cycle.

[9] Perl A.K., Wilgenbus P., Dahl U., Semb H., Christofori G. (1998). A causal role for E-cadherin in the transition from adenoma to carcinoma. Nature.

[10] Christofori G., Semb H. (1999). The role of the cell-adhesion molecule E-cadherin as a tumour-suppressor gene. Trends Biochem. Sci..

[11] Gilles C., Polette M., Mestdagt M., Nawrocki-Raby B., Ruggeri P., Birembaut P., Foidart J.M. (2003). Transactivation of vimentin by b-catenin in human breast cancer cells. Cancer Res..

[12] Auersperg N., Pan J., Grove B.D., Peterson T., Fisher J., Maines-Bandiera S., Somasiri A., Roskelley C.D. (1999). E-cadherin induces mesenchymal-to-epithelial transition in human ovarian surface epithelium. Proc. Natl. Acad. Sci. USA.

[13] Hay E.D., Zuk A. (1995). Transformations between epithelium and mesenchyme: Normal, pathological, and experimentally induced. Am. J. Kidney Dis..

[14] Vleminckx K., Vakaet L., Mareel M., Fiers W., van Roy F. (1991). Genetic manipulation of E-cadherin expression by epithelial tumor cells reveals an invasion suppressor role. Cell.

[15] Takeichi M. (1993). Cadherins in cancer: Implications for invasion and metastasis. Curr. Opin. Cell Biol..

[16] Nieman M.T., Prudoff R.S., Johnson K.R., Wheelock M.J. (1999). N-cadherin promotes motility in human breast cancer cells regardless of their E-cadherin expression. J. Cell Biol..

[17] Vestweber D. (2008). VE-cadherin: The major endothelial adhesion molecule controlling cellular junctions and blood vessel formation. Arter. Thromb. Vasc. Biol..

[18] Rezaei M., Cao J., Friedrich K., Kemper B., Brendel O., Grosser M., Adrian M., Baretton G., Breier G., Schnittler H.J. (2018). The expression of VE-cadherin in breast cancer cells modulates cell dynamics as a function of tumor differentiation and promotes tumor-endothelial cell interactions. Histochem. Cell Biol..

[19] Labelle M., Schnittler H.J., Aust D.E., Friedrich K., Baretton G., Vestweber D., Breier G. (2008). Vascular endothelial cadherin promotes breast cancer progression via transforming growth factor b signaling. Cancer Res..

[20] Cavaco A., Rezaei M., Niland S., Eble J.A. (2017). Collateral Damage Intended-Cancer-Associated Fibroblasts and Vasculature Are Potential Targets in Cancer Therapy. Int. J. Mol. Sci..

[21] Eble J.A., Niland S. (2019). The extracellular matrix in tumor progression and metastasis. Clin. Exp. Metastasis.

[22] Kikuchi S., Yoshioka Y., Prieto-Vila M., Ochiya T. (2019). Involvement of Extracellular Vesicles in Vascular-Related Functions in Cancer Progression and Metastasis. Int. J. Mol. Sci..

[23] van Niel G., D’Angelo G., Raposo G. (2018). Shedding light on the cell biology of extracellular vesicles. Nat. Rev. Mol. Cell Biol..

[24] Zhang X., Yuan X., Shi H., Wu L., Qian H., Xu W. (2015). Exosomes in cancer: Small particle, big player. J. Hematol. Oncol..

[25] Feng D., Zhao W.L., Ye Y.Y., Bai X.C., Liu R.Q., Chang L.F., Zhou Q., Sui S.F. (2010). Cellular internalization of exosomes occurs through phagocytosis. Traffic.

[26] Aga M., Bentz G.L., Raffa S., Torrisi M.R., Kondo S., Wakisaka N., Yoshizaki T., Pagano J.S., Shackelford J. (2014). Exosomal HIF1alpha supports invasive potential of nasopharyngeal carcinoma-associated LMP1-positive exosomes. Oncogene.

[27] Maji S., Chaudhary P., Akopova I., Nguyen P.M., Hare R.J., Gryczynski I., Vishwanatha J.K. (2017). Exosomal Annexin II Promotes Angiogenesis and Breast Cancer Metastasis. Mol. Cancer Res..

[28] Zeng Y., Yao X., Liu X., He X., Li L., Liu X., Yan Z., Wu J., Fu B.M. (2019). Anti-angiogenesis triggers exosomes release from endothelial cells to promote tumor vasculogenesis. J. Extracell. Vesicles.

[29] Maishi N., Annan D.A., Kikuchi H., Hida Y., Hida K. (2019). Tumor Endothelial Heterogeneity in Cancer Progression. Cancers (Basel).

[30] Maniotis A.J., Folberg R., Hess A., Seftor E.A., Gardner L.M., Pe’er J., Trent J.M., Meltzer P.S., Hendrix M.J. (1999). Vascular channel formation by human melanoma cells in vivo and in vitro: Vasculogenic mimicry. Am. J. Pathol..

[31] Chang Y.S., di Tomaso E., McDonald D.M., Jones R., Jain R.K., Munn L.L. (2000). Mosaic blood vessels in tumors: Frequency of cancer cells in contact with flowing blood. Proc. Natl. Acad. Sci. USA.

[32] Tian X., Liu Z., Niu B., Zhang J., Tan T.K., Lee S.R., Zhao Y., Harris D.C., Zheng G. (2011). E-cadherin/b-catenin complex and the epithelial barrier. J. Biomed. Biotechnol..

[33] Ramos T.L., Sanchez-Abarca L.I., Muntion S., Preciado S., Puig N., Lopez-Ruano G., Hernandez A., Redondo A., Ortega R., Rodriguez C. (2016). MSC surface markers (CD44, CD73, and CD90) can identify human MSC-derived extracellular vesicles by conventional flow cytometry. Cell Commun. Signal..

[34] Viaud M., Ivanov S., Vujic N., Duta-Mare M., Aira L.E., Barouillet T., Garcia E., Orange F., Dugail I., Hainault I. (2018). Lysosomal Cholesterol Hydrolysis Couples Efferocytosis to Anti-Inflammatory Oxysterol Production. Circ. Res..

[35] Serizier S.B., McCall K. (2017). Scrambled Eggs: Apoptotic Cell Clearance by Non-Professional Phagocytes in the Drosophila Ovary. Front. Immunol..

[36] Fornetti J., Flanders K.C., Henson P.M., Tan A.C., Borges V.F., Schedin P. (2016). Mammary epithelial cell phagocytosis downstream of TGF-b3 is characterized by adherens junction reorganization. Cell Death Differ..

[37] Wheelock M.J., Shintani Y., Maeda M., Fukumoto Y., Johnson K.R. (2008). Cadherin switching. J. Cell Sci..

[38] Dai X., Cheng H., Bai Z., Li J. (2017). Breast Cancer Cell Line Classification and Its Relevance with Breast Tumor Subtyping. J. Cancer.

[39] Navarro P., Ruco L., Dejana E. (1998). Differential localization of VE- and N-cadherins in human endothelial cells: VE-cadherin competes with N-cadherin for junctional localization. J. Cell Biol..

[40] Sigurdsson V., Hilmarsdottir B., Sigmundsdottir H., Fridriksdottir A.J., Ringner M., Villadsen R., Borg A., Agnarsson B.A., Petersen O.W., Magnusson M.K. (2011). Endothelial induced EMT in breast epithelial cells with stem cell properties. PLoS ONE.

[41] Maeda M., Johnson E., Mandal S.H., Lawson K.R., Keim S.A., Svoboda R.A., Caplan S., Wahl J.K., Wheelock M.J., Johnson K.R. (2006). Expression of inappropriate cadherins by epithelial tumor cells promotes endocytosis and degradation of E-cadherin via competition for p120(ctn). Oncogene.

[42] Brasch J., Katsamba P.S., Harrison O.J., Ahlsen G., Troyanovsky R.B., Indra I., Kaczynska A., Kaeser B., Troyanovsky S., Honig B. (2018). Homophilic and Heterophilic Interactions of Type II Cadherins Identify Specificity Groups Underlying Cell-Adhesive Behavior. Cell Rep..

[43] Delgado-Bellido D., Serrano-Saenz S., Fernandez-Cortes M., Oliver F.J. (2017). Vasculogenic mimicry signaling revisited: Focus on non-vascular VE-cadherin. Mol. Cancer.

[44] O’Loghlen A. (2018). Role for extracellular vesicles in the tumour microenvironment. Philos. Trans. R. Soc. B Biol. Sci..

[45] Margolis L., Sadovsky Y. (2019). The biology of extracellular vesicles: The known unknowns. PLoS Biol..

[46] Tong Y., Zhou Y.L., Wang Y.X., Zhao P.Q., Wang Z.Y. (2016). Retinal pigment epithelium cell-derived exosomes: Possible relevance to CNV in wet-age related macular degeneration. Med. Hypotheses.

[47] Strilic B., Yang L., Albarran-Juarez J., Wachsmuth L., Han K., Muller U.C., Pasparakis M., Offermanns S. (2016). Tumour-cell-induced endothelial cell necroptosis via death receptor 6 promotes metastasis. Nature.

[48] Flemming S., Burkard N., Renschler M., Vielmuth F., Meir M., Schick M.A., Wunder C., Germer C.T., Spindler V., Waschke J. (2015). Soluble VE-cadherin is involved in endothelial barrier breakdown in systemic inflammation and sepsis. Cardiovasc. Res..

[49] Kleine-Bruggeney H., van Vliet L.D., Mulas C., Gielen F., Agley C.C., Silva J.C.R., Smith A., Chalut K., Hollfelder F. (2019). Long-Term Perfusion Culture of Monoclonal Embryonic Stem Cells in 3D Hydrogel Beads for Continuous Optical Analysis of Differentiation. Small.

[50] Kleine-Brueggeney H., Zorzi G.K., Fecker T., El Gueddari N.E., Moerschbacher B.M., Goycoolea F.M. (2015). A rational approach towards the design of chitosan-based nanoparticles obtained by ionotropic gelation. Colloids Surf. B Biointerfaces.

[51] Cavaco A.C.M., Rezaei M., Caliandro M.F., Lima A.M., Stehling M., Dhayat S.A., Haier J., Brakebusch C., Eble J.A. (2018). The Interaction between Laminin-332 and α3β1 Integrin Determines Differentiation and Maintenance of CAFs, and Supports Invasion of Pancreatic Duct Adenocarcinoma Cells. Cancers (Basel).

[52] Rezaei M., Cavaco A.C., Seebach J., Niland S., Zimmermann J., Hanschmann E.M., Hallmann R., Schillers H., Eble J.A. (2019). Signals of the Neuropilin-1-MET Axis and Cues of Mechanical Force Exertion Converge to Elicit Inflammatory Activation in Coherent Endothelial Cells. J. Immunol..

[53] Livak K.J., Schmittgen T.D. (2001). Analysis of relative gene expression data using real-time quantitative PCR and the 2(-Delta Delta C(T)) Method. Methods.

[54] Guo S., Lok J., Liu Y., Hayakawa K., Leung W., Xing C., Ji X., Lo E.H. (2014). Assays to examine endothelial cell migration, tube formation, and gene expression profiles. Methods Mol. Biol..

