# Supplementary material for: Extracellular Vesicle Transfer from Endothelial Cells Drives VE-Cadherin Expression in Breast Cancer Cells, Thereby Causing Heterotypic Cell Contacts
Source: Cancers (Basel). 2020 Aug 1;12(8):2138. doi: 10.3390/cancers12082138 (PMC7463713; doi:10.3390/cancers12082138)
Supplement: Supplementary file 1 [file cancers-12-02138-s001.pdf]

Supplementary Materials

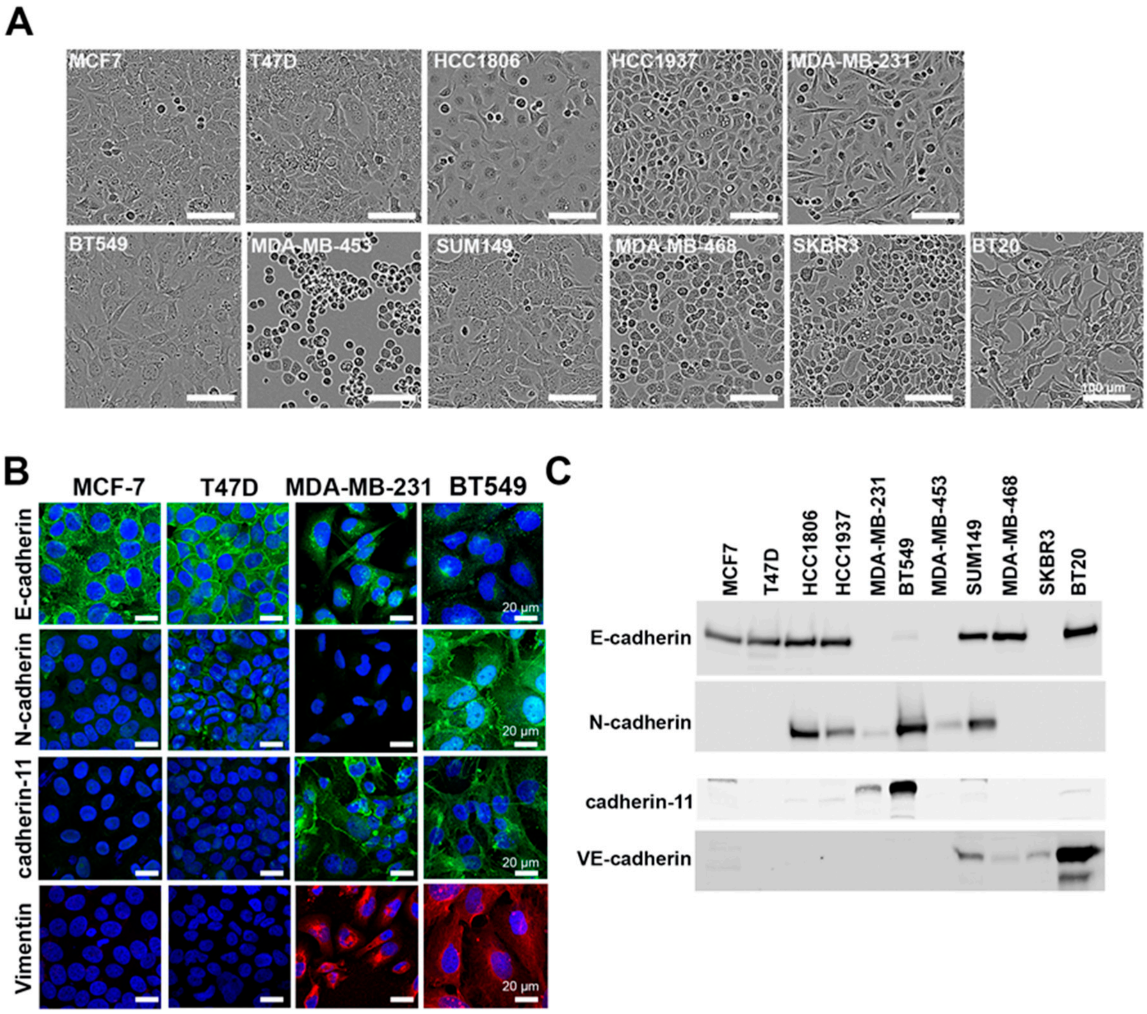

Figure S1. Characterization of different breast cancer cell lines. (**A**) Representative bright-field images of eleven breast cancer lines (MCF7, T47D, HCC1806, HCC1937, MDA-MB-231, BT549, MDA-MB-453, SUM149, MDA-MB-468, SKBR3, and BT20). (**B**) Immunofluorescence staining and (**C**) western blot analysis of E-cadherin, N-cadherin, cadherin-11, and VE-cadherin in these breast cell lines. An equal concentration of samples (50 μg) were loaded onto polyacrylamide gels. Immunofluorescent detection of vimentin was used to identify invasive breast cancer cell lines. Nuclei are stained in blue, cadherins in green, and vimentin in red.

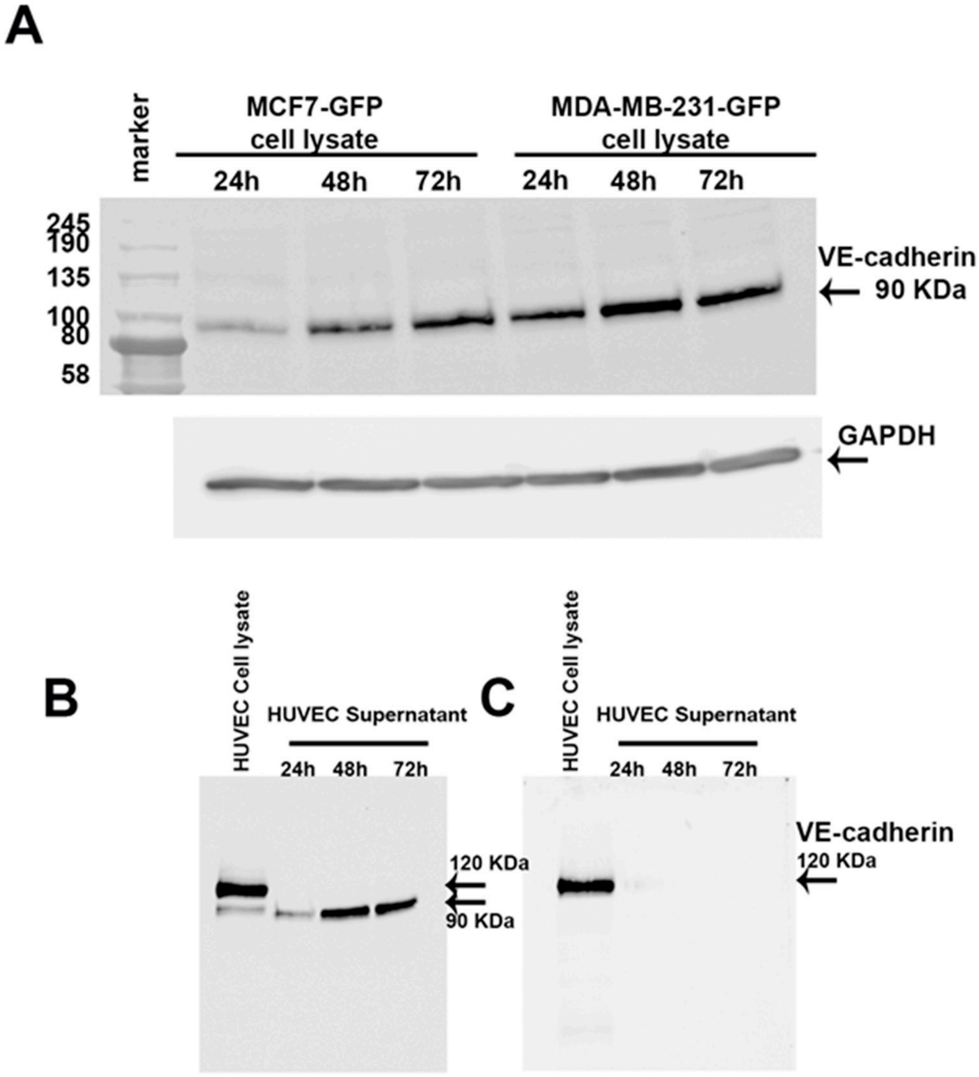

Figure S2. The soluble VE-cadherin ectodomain, termed sVE-cadherin, was detected in cancer cells treated with HUVEC supernatant. (**A**) MCF7-GFP and MDA-MB-231-GFP cells were treated with HUVEC-conditioned medium for different times (24, 48, and 72 h), thereafter lysed and tested for VE-cadherin expression by western blot with an antibody directed against the VE-cadherin extracellular domain (BV9). (**B**) Western blot analysis of conditioned medium (CM) collected from HUVECs with VE-cadherin (BV9) antibody after 24, 48, and 72 h revealed an accumulation of sVE-cadherin in a time-dependent manner. (**C**) VE-cadherin could not be detected in the HUVEC CM by using an antibody against the intracellular domain of VE-cadherin (C-19).

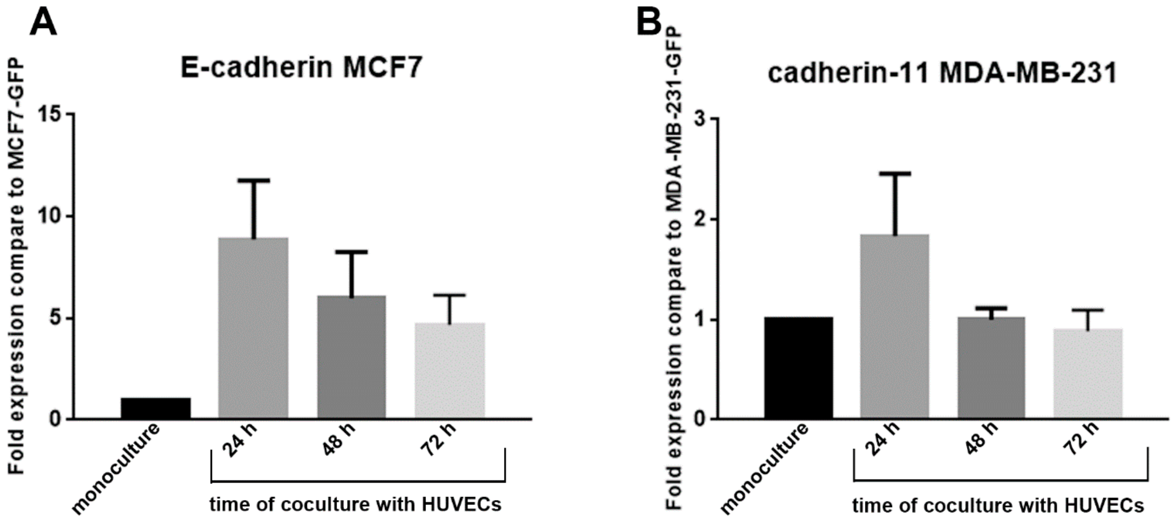

Figure S3. mRNA levels of endogenous cadherin changed in MCF7 and MDA-MB-231 cells after co-culturing with HUVECs. (**A**) MCF7-GFP and (**B**) MDA-MB-231-GFP cells co-cultured with HUVECs over different time points (24, 48, and 72 h) were isolated by FACS and lysed to quantify the E-cadherin and cadherin-11 mRNA levels in MCF7 and MDA-MB-231 cells, respectively. The real-time PCR data were normalized to the level of TOP1 mRNA, used as an internal control. For evaluation of E-cadherin mRNA level, the numbers of samples analyzed were: n = 3 MCF7 monoculture and for co-culture n = 5 at 24 h, n = 3 at 48 h, and n = 5 at 72 h. The numbers of samples analyzed for the cadherin-11 mRNA level were n = 3.

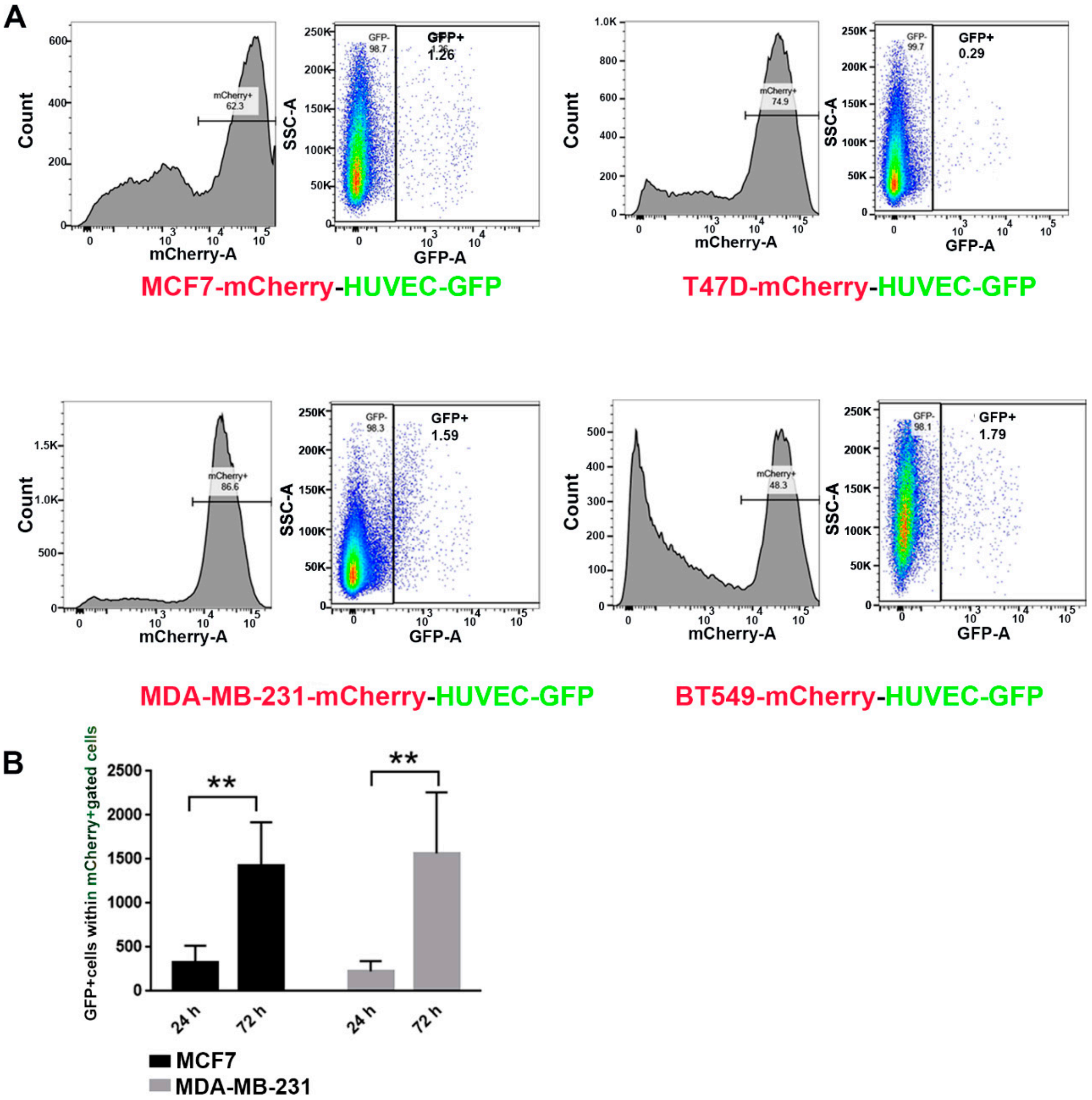

Figure S4. GFP labeled EVs of HUVECs are transferred from ECs to TCs. (**A**) Different breast cancer cell lines (MCF7-mCherry, T47D-mCherry, MDA-MB-231-mCherry, and BT549-mCherry) were co-cultured with GFP-labeled HUVECs for 72 h and analyzed for GFP+ cells within the mCherry+-gated cancer cells by flow cytometry. (**B**) The graph shows the number of mCherry+/ GFP+ cells after 24 h and 72 h, for the calculation of double-positive cells n=4 MCF7 cells and n=5 MDA-MB-231 cells. Mean values ± SD are shown (**, $p \leq 0.01$).

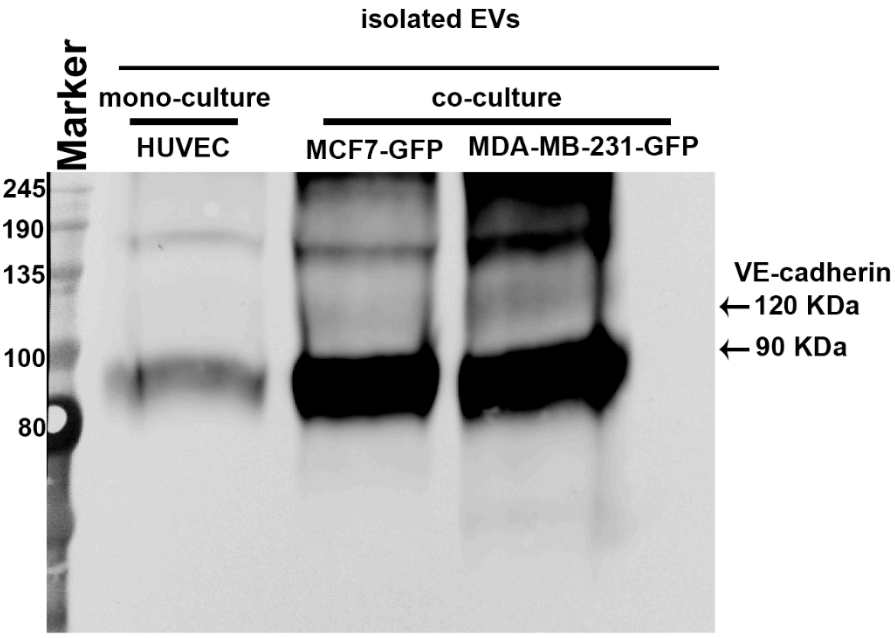

Figure S5. TCs influence the contents of EVs secreted by HUVECs. Western blot analysis showed the presence of full-length VE-cadherin fragment only in the EVs that were isolated from co-culture supernatant. The EVs were isolated from cell supernatants of monocultured HUVECs and of cocultures of HUVECs with GFP-tagged MCF-7 and MDA-MB231 cells, by utilizing the exosome isolation kit (Exo-spin, Cell Guidance Systems).

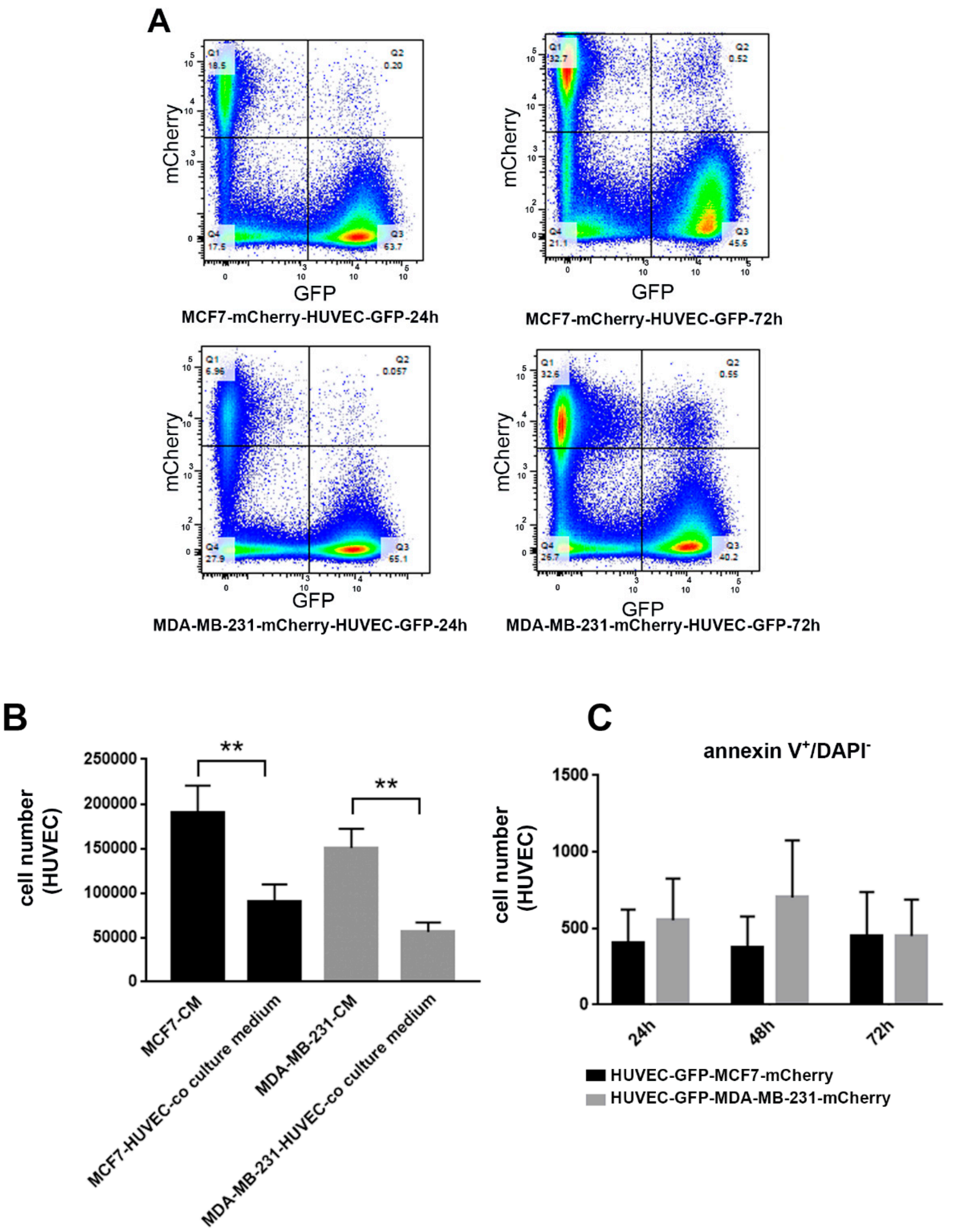

Figure S6. Cancer cells induce cell death in HUVECs, but HUVECs promote cancer cell proliferation via direct cell-cell interaction. (**A**) After 24 and 72 h of co-culturing, the number of GFP-labeled HUVECs and mCherry-labeled cancer cells, MCF7 or MDA-MB-231, were analyzed by flow cytometry. (**B**) The proliferation of HUVECs treated with conditioned medium of a TC-EC co-culture was analyzed by using flow cytometry. Mean values ± SD are shown (**, $p \leq 0.01$). (**C**) HUVECs co-cultured with TCs were stained with annexin V-Alexa Fluor-647, and with DAPI for quantification of apoptotic cells (Annexin V/Alexa Fluor-647+) or dead cells (DAPI+) by flow cytometry. The graph represents the analyzed flow cytometric data of 3 independent experiments.

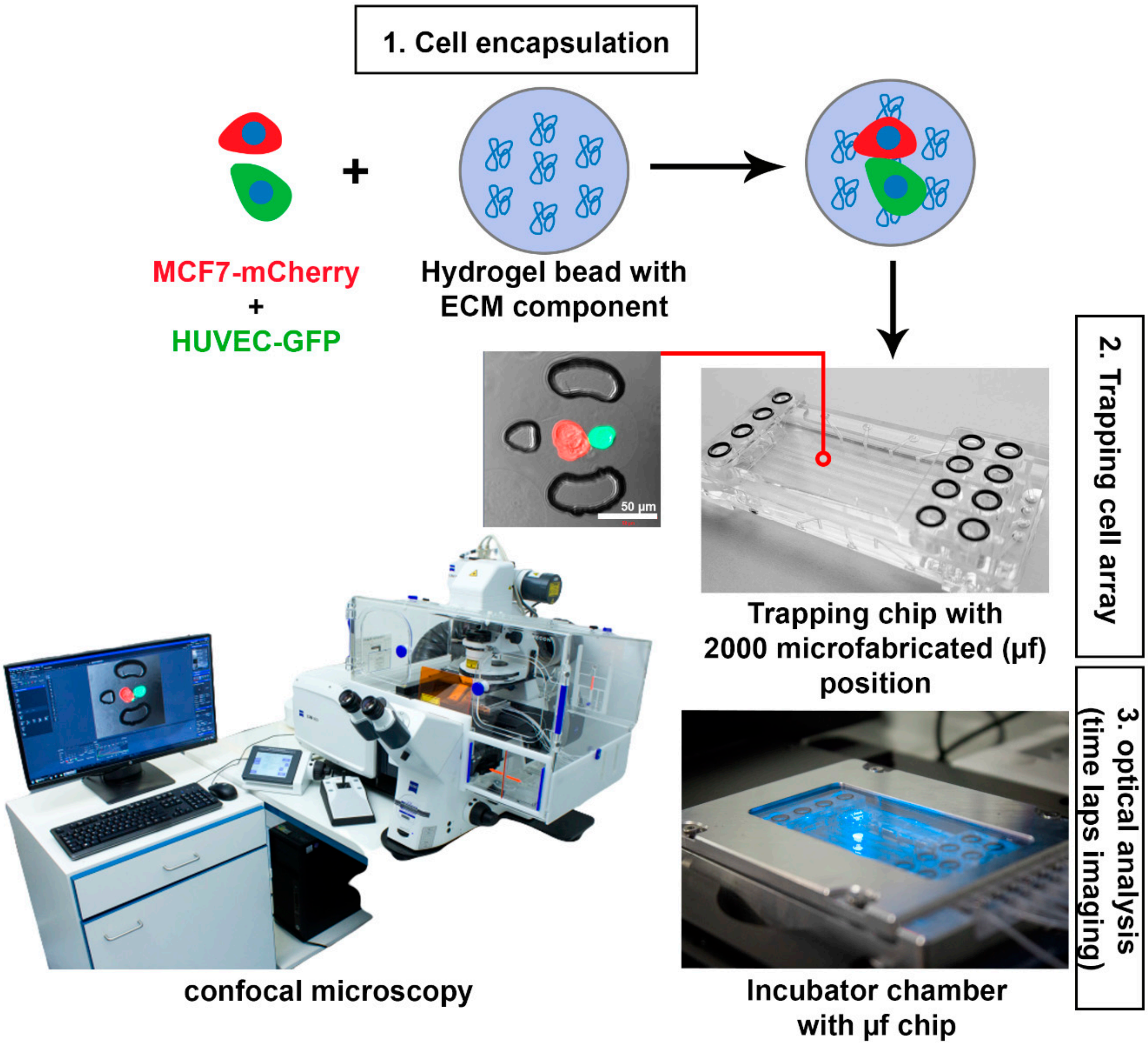

Figure S7. Scheme of cell-cell interaction at single-cell resolution in 3D environment. HUVEC (green) and MCF7 (red) cell suspensions were mixed in PBS. Agarose droplets containing cells were generated in a bead formation chip by using syringe pumps. After agarose droplets gelation, beads containing cells were transferred and immobilized into a trapping chip by using a control unit (EVORION Biotechnologies). TC-EC interaction was monitored by time-lapse confocal microscopy (LSM800), at 37C°, with 10× magnification.

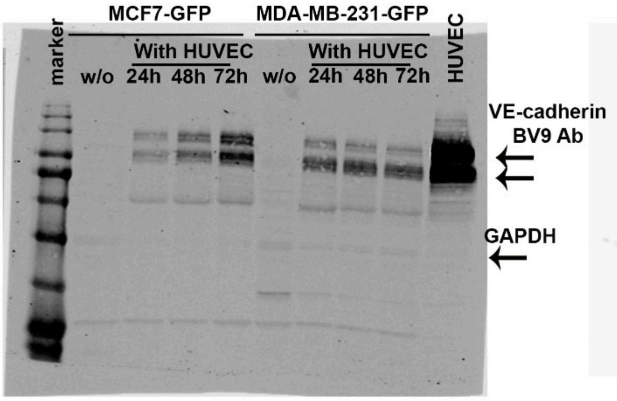

Fig. 1 B

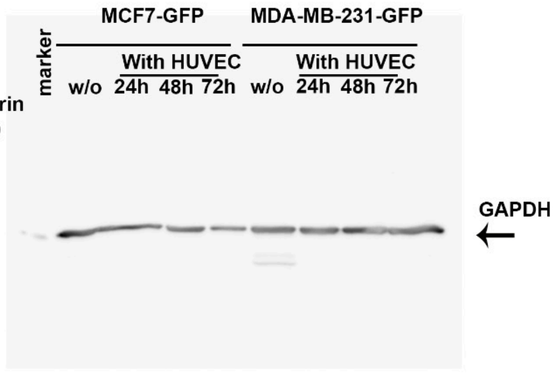

Fig. 1 B

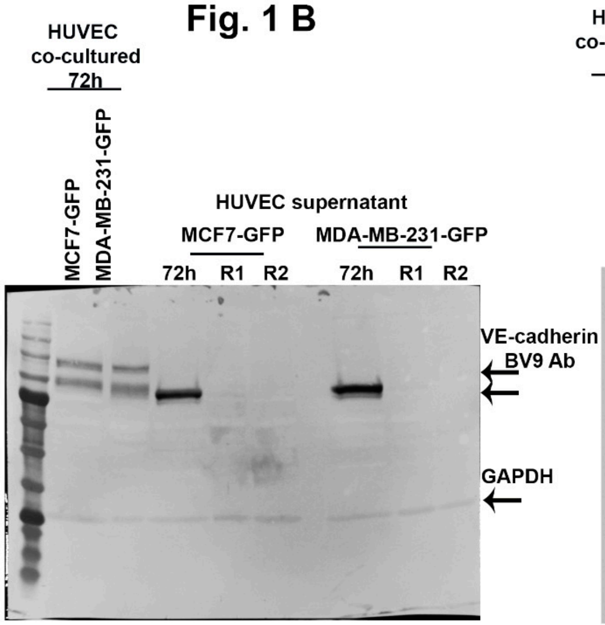

Fig. 1 E

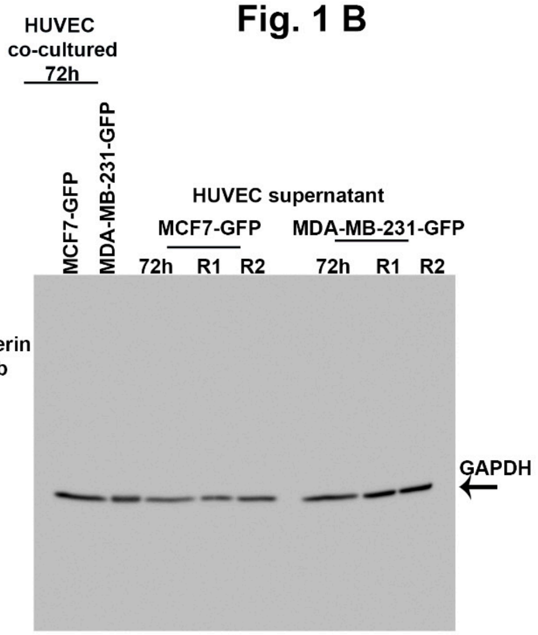

Fig. 1 E

Figure S8. Western blots to figure 1B and 1E.

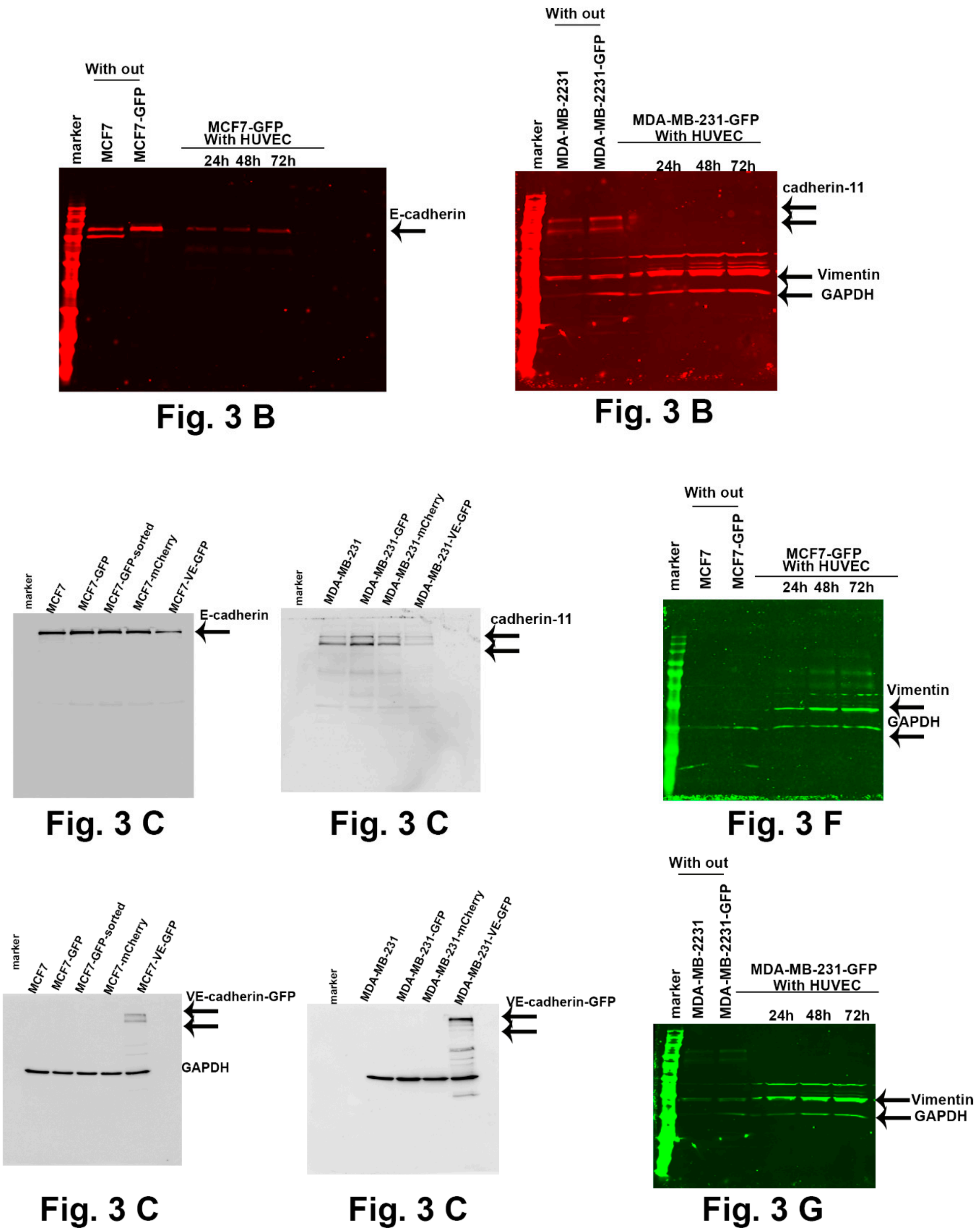

Figure S9. Western blots to figure 3B, 3C, 3F and 3G.

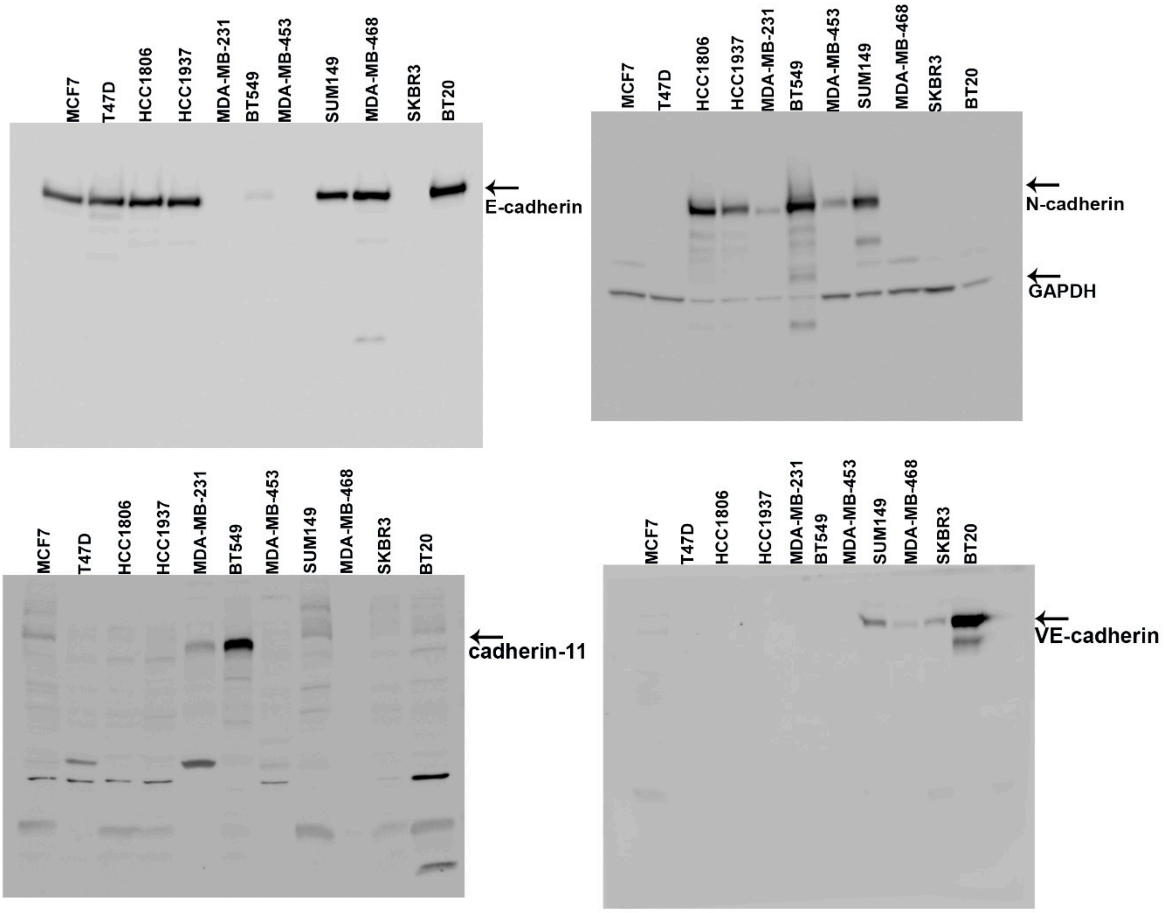

Figure S10. Western blots of figure 1C.

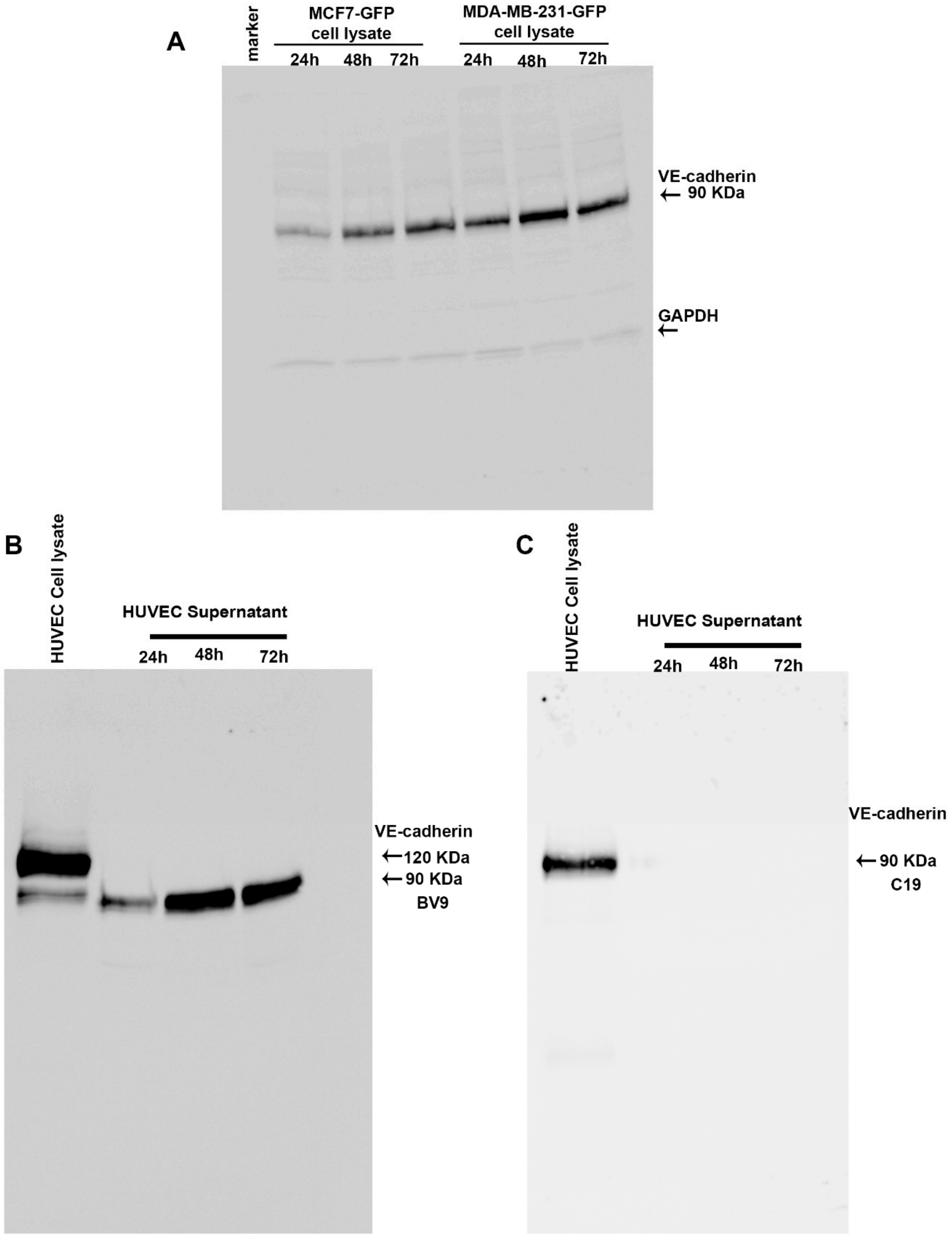

Figure S11. Western blots of figure S2.

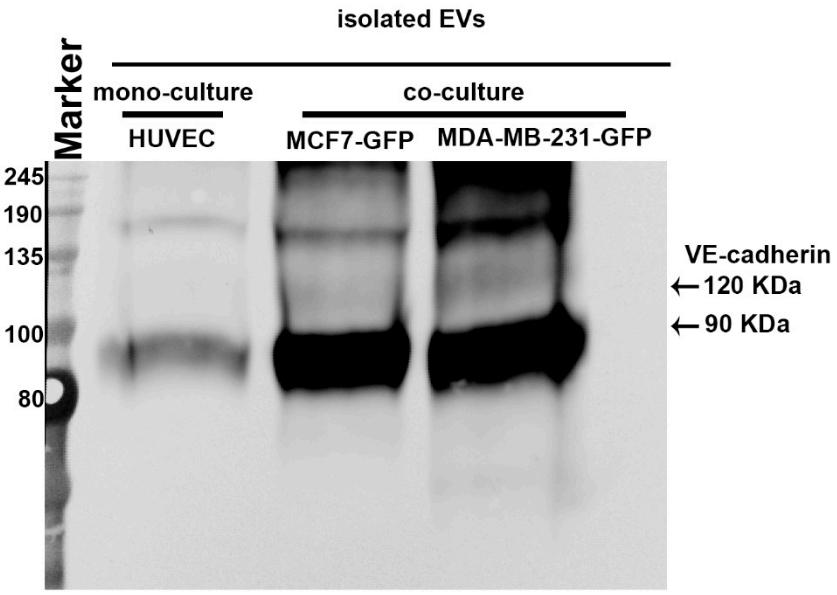

Figure S12. Western blots of figure S5.
